# The Effectiveness of a Traditional Chinese Medicine–Based Mobile Health App for Individuals With Prediabetes: Randomized Controlled Trial

**DOI:** 10.2196/41099

**Published:** 2023-06-20

**Authors:** Hsueh-Wen Chung, Chen-Jei Tai, Polun Chang, Wen-Lin Su, Li-Yin Chien

**Affiliations:** 1 Department of Nursing College of Nursing National Yang Ming Chiao Tung University Taipei City Taiwan; 2 Tai's Traditional Chinese Medicine Clinic Taipei City Taiwan; 3 Department of Obstetrics and Gynecology School of Medicine, College of Medicine Taipei Medical University Taipei City Taiwan; 4 Institute of Biomedical Informatics National Yang Ming Chiao Tung University Taipei City Taiwan; 5 Division of Pulmonary and Critical Care Medicine Department of Internal Medicine Taipei Tzu Chi Hospital, Buddhist Tzu Chi Medical Foundation New Taipei City Taiwan; 6 School of Medicine Tzu Chi University Hualien Taiwan; 7 Institute of Community Health Care College of Nursing National Yang Ming Chiao Tung University Taipei City Taiwan

**Keywords:** mHealth app, prediabetes, traditional Chinese medicine, health-related quality of life, body constitution, meridian energy

## Abstract

**Background:**

Traditional Chinese medicine (TCM) theories assert that body constitution and meridian energy lay the foundation for disease prevention. TCM-based health concepts have not yet been incorporated into mobile health (mHealth) apps for individuals with prediabetes.

**Objective:**

The aim of this study was to examine the effectiveness of a TCM mHealth app for individuals with prediabetes.

**Methods:**

This randomized controlled trial recruited 121 individuals with prediabetes at a teaching hospital in New Taipei City between February 2020 and May 2021. The participants were randomly assigned to the TCM mHealth app group (n=42), ordinary mHealth app group (n=41), or control group (n=38). All participants received the usual care that included 15-20 minutes of health education about the disease, along with healthy diet and exercise encouragement. The ordinary mHealth app included physical activity (PA), diet, and disease education, along with individual records. The TCM mHealth app additionally included *qi* and body constitution information, along with constitution-based PA and diet advice. The control group received the usual care alone and did not have access to any app. Data were collected at baseline, at the end of the 12-week intervention, and 1 month after the intervention. Body constitution, including *yang-*deficiency, *yin-*deficiency, and phlegm-stasis, was measured according to the Body Constitution Questionnaire, with higher scores indicating a greater deficiency. Body energy was examined using the Meridian Energy Analysis Device. The Short-Form 36 questionnaire was used to evaluate health-related quality of life (HRQOL), which yielded physical component scores and mental component scores, with higher scores indicating better physical and mental aspects of HRQOL, respectively.

**Results:**

Compared to the control group, the TCM mHealth app group showed greater improvement in hemoglobin A_1c_ (HbA_1c_), *yang*-deficiency and phlegm-stasis body constitution, and BMI; however, no significant differences were found in these outcomes between the TCM mHealth app and ordinary mHealth app groups. The TCM mHealth app group showed better improvement in body energy and mental component scores than the ordinary mHealth app group. There were no significant differences in fasting plasma glucose, *yin*-deficiency body constitution, Dietary Approaches to Stop Hypertension dietary behavior, and total PA among the three groups after the intervention.

**Conclusions:**

Use of either the ordinary or TCM mHealth app improved HRQOL among individuals with prediabetes. Compared to the outcomes of controls not using any app, use of the TCM mHealth app was effective at improving HbA_1c_, BMI, *yang*-deficiency and phlegm-stasis body constitution, and HRQOL. Moreover, using the TCM mHealth app seemed to improve the body energy and HRQOL more than when using the ordinary mHealth app. Further studies with a larger sample size and longer follow-up period may be necessary to determine whether the differences favoring the TCM app are clinically meaningful.

**Trial Registration:**

ClinicalTrials.gov NCT04096989; https://clinicaltrials.gov/ct2/show/NCT04096989

## Introduction

Prediabetes is a subhealth condition characterized by higher than normal blood sugar levels, but not yet at a sufficiently high level to warrant a diagnosis of type 2 diabetes mellitus (T2DM) [1]. The American Diabetes Association proposes a diagnosis of prediabetes according to a fasting plasma glucose (FPG) level in the range of 100-125 mg/dL, hemoglobin A_1c_ (HbA_1c_) in the range of 5.7%-6.4%, or 2-hour postprandial blood glucose level after the 75-g oral glucose test in the range of 140-199 mg/dL [2]. The prevalence of prediabetes among adults has been estimated to be 34.5% [3], with approximately 5%-10% of those diagnosed with prediabetes ultimately developing T2DM within 1 year [4].

The Centers for Disease Control and Prevention Diabetes Prevention Program (DPP) has been shown to effectively delay or prevent the development of T2DM among individuals diagnosed with prediabetes [5-7]. To reduce the cost and promote DPP-based lifestyle interventions, a technology-assisted DPP intervention is advised to be adopted with mobile and web-based apps and text messaging. Using a smartphone and computer could be ideal techniques to create widely available, easy-to-use diabetes prevention tools [8,9]. A randomized controlled trial (RCT) showed that mobile-delivered DPP achieved significant weight and BMI reductions compared with usual care. However, the intervention does not appear to be effective in controlling HbA_1c_ [10]. Another RCT developed a fully automated algorithm-driven behavioral intervention delivered via the web, internet, mobile phone, and automated phone calls, demonstrating that the intervention group had significantly decreased FPG and HbA_1c_, and increased physical activity (PA) and vegetable consumption [11,12].

People’s lifestyles and behaviors have close associations with their sociocultural background [13]. Body constitution and meridian energy are fundamental concepts in traditional Chinese medicine (TCM). Body constitution represents the individual’s body condition that makes them susceptible to certain diseases but not others [14]. Body constitution forms the basis for disease treatment and prevention in TCM [15]. Meridians are channels that form a network in the body through which *qi* and blood (vital energy) flow [16,17]. The energy flow throughout the body via the 12 meridians is referred to as the meridian energy [18]. A high mean meridian energy (the average of the 12 meridian energies or body energy) usually means that *qi* and blood flow are strong and move smoothly throughout the meridians [19].

Several measures use different types of classifications for body constitution [20-23]. Nevertheless, *yang*-deficiency, *yin*-deficiency, and phlegm-stasis body constitution are common features that are prevalent in patients with chronic diseases [24,25]. *Yang-*deficiency refers to an insufficiency of *qi*. Individuals with this deficiency may experience symptoms such as fatigue, shortness of breath, chills, loose stool, and a large volume of urine. *Yin-*deficiency reflects an insufficiency in blood and interstitial fluids, and thus patients with *yin-* deficiency may experience symptoms such as being constantly thirsty, experiencing hot flushes, hard stool, and a low volume of urine. Phlegm is a viscous and turbid pathological factor formed due to an imbalance in body fluid. Phlegm-stasis refers to the accumulation of phlegm in the body as a form of condensation, which results in dizziness, chest tightness, and numbness in the limbs [26]. These TCM concepts of blood, phlegm, and fluids are not equivalent to the Western uses of these terms, but are instead used to represent energetic qualities. For example, in TCM, blood is considered a vehicle for *qi* that carries inherent energy; it nourishes and moistens the body, and it circulates nutritive *qi*. The definitions of relevant TCM terms are presented in Multimedia Appendix 1 [14,16,17,20-22,26-31].

Previous studies showed that individuals with prediabetes or diabetes were more deficient in the body constitutions of *yang-*deficiency, *yin-*deficiency, and phlegm-stasis, and also had lower meridian energy [20,21,32]. In addition, body constitution is related to an unhealthy lifestyle, in which *yang-*deficiency and phlegm-stasis are related to physical inactivity and smoking, respectively [33]. Despite high heterogeneity in the contents of available interventions, TCM lifestyle programs typically involve body constitution–based TCM health education, Chinese dietary therapy, and traditional Chinese exercises [34-38]. These programs were designed to stimulate the *qi*-blood circulation and regulate *Zang-Fu* to enhance quality of life. Previous studies have shown that TCM lifestyle programs improve body constitution [34], dietary behavior [35], and PA [36], while helping to lower blood sugar [37,38]. According to TCM theory, an improved body constitution could decrease the susceptibility to chronic diseases [14]. Increased body energy would manifest in better stamina through TCM lifestyle programs [17]. Therefore, we hypothesized that body constitution and body energy could be improved by body constitution and TCM- based lifestyle modification through *Qigong* (a type of PA) and a healthy dietary regimen, and thus help to achieve blood sugar control and enhance health.

To the best of our knowledge, no study has been conducted to identify whether TCM-based health concepts could be incorporated into a mobile health (mHealth) app for individuals with prediabetes. The need for incorporating TCM body constitutions is based on two key factors: (1) as a sociocultural appropriate method to contextualize PA and diet, and (2) as possible mediation variables for blood sugar control such as HbA_1c_ and FBG. Accordingly, the aim of this study was to develop a TCM mHealth app and examine its effectiveness on blood sugar control, body constitution, body energy, and health-related quality of life (HRQOL) as primary outcomes, as well as on BMI, dietary behavior, and PA as secondary outcomes among individuals with prediabetes. The hypothesis was that the TCM mHealth app would improve overall health through modifying health behavior and BMI. Therefore, the primary outcomes were overall health indicators (including body constitution and meridian energy) and the secondary outcomes were BMI and health behaviors.

## Methods

### Study Design

This study was an open-label, parallel-group RCT (ClinicalTrials.gov NCT04096989) with a three-group design. We cooperated with the health examination center and outpatient clinics at a teaching hospital in northern Taiwan to recruit individuals diagnosed with prediabetes from February 2020 to May 2021. The inclusion criteria were (1) having been diagnosed with prediabetes (according to an HbA_1c_ of 5.7%-6.4% or an FPG level of 100-125 mg/dL [2]); (2) aged 20 years and above; (3) not having cardiopulmonary disease, cancer, or other major diseases; and (4) provision of informed consent. Those who had used hypoglycemic agents, β-blockers, thiazide diuretics, nicotinic acid, or steroids within the past 3 months were excluded.

Participants were randomly assigned to three groups: TCM mHealth app, ordinary mHealth app, or control group. A statistician drew up a computer-generated randomization list. The allocation sequence was kept in an opaque, sealed, and stapled envelope, and a staff member in the outpatient clinic who was not involved in the study held the sealed envelopes. After the participants agreed to participate in the study, the researcher opened the envelope to reveal their group assignment.

The informed consent form was signed by all participants before enrollment in the study. The informed consent form stated that the risk of participation in this study was low. If the participants felt physically or mentally unwell due to their participation, they had to contact the researchers and had the right to withdraw at any time. No adverse events were reported during the study period.

### Ethics Approval

This study was approved by the institutional review board at Taipei Tzu Chi Hospital (approval no. 08-X-026).

### Participants

The required sample size was calculated on the basis of repeated-measures ANOVA (α=.05, power=0.80, effect size=0.3) with three repeated measurements as per a previous study [37]. G-power software indicated that the required sample size was 31 per group. To account for 30% attrition [39], we recruited 121 participants in the study.

**Figure 1:**
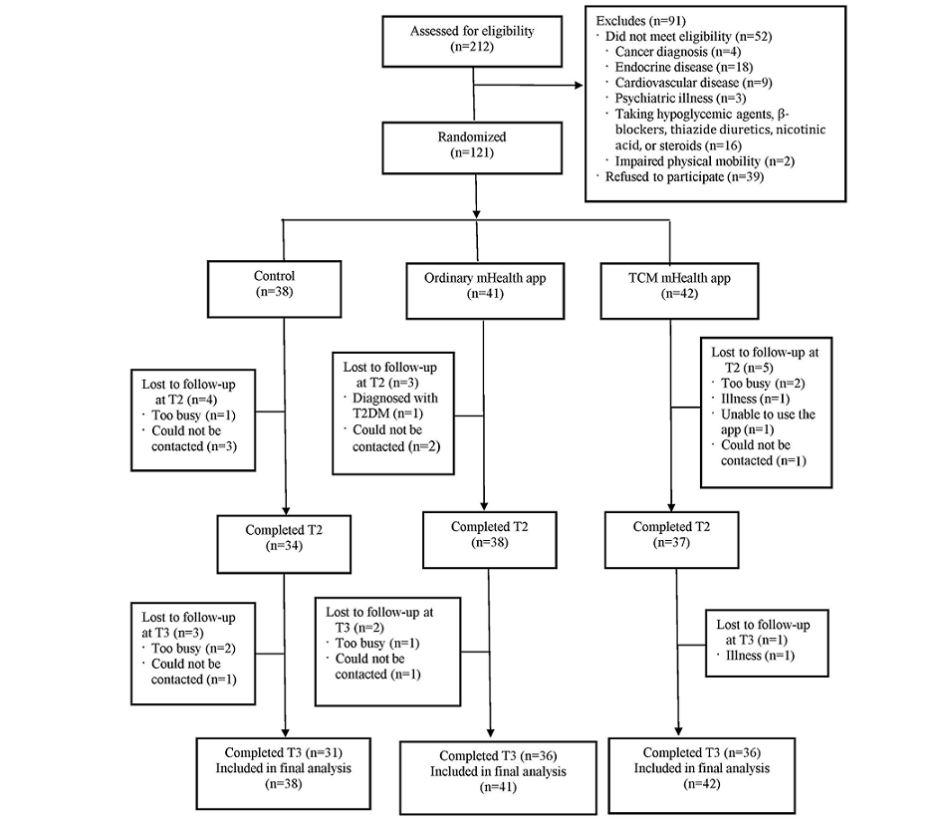
Participant flow diagram. mHealth: mobile health; T1: baseline; T2: end of the intervention; T2DM: type 2 diabetes mellitus; T3: 1 month after the intervention; TCM: traditional Chinese medicine.

Figure 1 presents a flow diagram of participant allocation to the three groups. A total of 212 individuals with prediabetes were assessed for eligibility, 52 of whom did not meet the eligibility criteria and 39 of whom declined to participate. A total of 121 participants were randomly assigned to the TCM mHealth app group (n=42), ordinary mHealth app group (n=41), or control group (n=38).

All participants received the usual care at the study hospital when they received the diagnosis of prediabetes. The usual care was 15-20 minutes of health education by family medicine physicians, including disease explanation, healthy diet advice, and exercise encouragement. The control group received usual care only without the use of any app.

### Intervention

An expert team that included nursing researchers, TCM doctors, Western medicine doctors, and app developers was formed to guide the development of the intervention app. The team decided that the content embedded in the app would be developed in accordance with the DPP [40,41] and a review of the literature [34,37,42]. The experts sketched, shared, and discussed potential app versions and developed prototypes. To gather user feedback on the prototypes, qualitative interviews with seven experts and five individuals were conducted, with an aim to determine user preference for the prototype and what could be improved in the app. We used the feedback to revise and finalize the mHealth app. We invited five individuals with prediabetes to use the TCM mHealth app for 30 minutes. Subsequently, a short questionnaire was administered to determine app usability and satisfaction. The participants reported positive experiences. They all agreed with the statements “I found the TCM mHealth app easy to use” and “I would recommend the TCM mHealth app to my friends and others.”

The mHealth app (both the ordinary and TCM versions) included four modules: health diary, health education, milestone, and chatroom. The “health diary” tracked the participant’s weight, BMI, blood sugar level, dietary diary, and PA over time. “Health education” provided information about specific topics such as learning about prediabetes, Dietary Approaches to Stop Hypertension (DASH) diet, and PA. The TCM mHealth app additionally included health education topics on body constitution, meridian energy, and advice on a body constitution–based diet (such as foods to avoid and foods that are recommended) and PA such as videos, pictures, and descriptive illustrations of types of Qigong (ie, Baduanjin and belly breath). A text message was sent to the participants to remind them to read the topics every week. Milestones were added for the participants to review their goals so that they could make adjustments to reach their monthly goals. For example, if the participants set a goal of an FPG level of 60-99 mg/dL, when this goal was achieved, a window would pop up to show the achievement. The participants could also check bar and line charts over 1 week or 1 month to compare the discrepancy between the actual and desired values as well as actual and ideal behaviors at different time points so that they could adjust their expected goal as needed.

In the “chatroom,” personal and group chat rooms were set up using the LINE app (Naver Corp, Gyeonggi Province, South Korea). The researchers sent text messages to the participants in the personal chat room, provided feedback on the results, and encouraged participants to share their experiences in the group chat room. The participants collected virtual gold by completing questionnaires and quizzes and by achieving the set goals. The participants could use the virtual gold to claim actual prizes from the researchers. Gamification elements were added to encourage engagement with the mHealth app. Screenshots and descriptions of the ordinary and TCM mHealth apps are presented in Multimedia Appendix 2. The taxonomy of behavior change techniques [43] used in the apps is presented in Multimedia Appendix 3.

The participants in the TCM and ordinary mHealth app groups received a face-to-face education session on how to use the mHealth app and create a user account. Both mHealth app groups received information about prediabetes and evidence-based methods to decrease the possibility of progression to T2DM (eg, moderate-intensity PA of ≥150 minutes/week, DASH diet, and disease health education). The TCM mHealth app group additionally received body constitution and *qi* information, as well as constitution-based PA and diet advice (see Multimedia Appendix 2). Participants who were assigned to the TCM mHealth app group filled out the Body Constitution Questionnaire (BCQ) [20-22] at the beginning of the study. Tailored TCM diet and PA advice based on the individual’s body constitution as determined by their BCQ results was incorporated into the TCM mHealth app.

The researchers monitored logins and log file analysis at least once every week at the mHealth app backend. If the participants did not use the app, complete the diary, or watch health education, additional text messages were sent to the participants. The CONSORT-EHEALTH guidelines [44] were followed in reporting this study (see Multimedia Appendix 4).

### Data Collection

Data were collected at baseline (T1), at the end of the 12-week intervention (T2), and 1 month after the intervention (T3). Sociodemographic characteristics (age, gender, marital status, education level, and employment status), clinical characteristics (history of chronic disease and use of TCM), and lifestyle factors (smoking and alcohol drinking) were collected by a structured questionnaire at baseline. Primary outcome measures, including blood sugar control (FPG and HbA_1c_ levels), body constitution, meridian energy, and HRQOL, were collected at T1, T2, and T3.

Body constitution was assessed by the BCQ [20-22], which measures the presence and severity of symptoms in the most recent 7 days. The BCQ consists of 44 symptom ratings rated on a 5-point Likert-type scale ranging from 1 (not at all) to 5 (very severe). The items were summed and categorized into three types of body constitutions: 19 items for *yang-*deficiency, 19 for *yin-*deficiency, and 16 for phlegm-stasis. A higher score indicates a higher level of deficiency in the body constitution type [45]. The reliability and validity of the BCQ have been supported by the results of previous studies [20-22].

Meridian energy was measured using the Meridian Energy Analysis Device (MEAD) ME-PRO 6.1.1 (Medpex Enterprise Ltd, Taichung, Taiwan). The level of meridian energy was assessed using the MEAD values for the 24 acupoints (Ryodoraku points) along the 12 meridians ranging from 0 to 200 µA [16,46]. The participants were required to take nothing by mouth for at least 8 hours; remove their shoes, socks, and metal materials that may cause a disturbance; and sit in the room for 10-15 minutes before the meridian energy checkup [47]. Body energy was yielded by the average of the energy flows through the 24 acupoints along the 12 meridians, which serves as an indicator for the fluency of *qi* and blood moving throughout the body. Individuals with prediabetes have a lower level of body energy [32]. A low level of body energy indicates that the meridians are blocked and thus the *qi* and blood circulation are not smooth. A lower level of body energy thus indicates worse stamina.

HRQOL was measured by the Medical Outcome Survey Short Form (SF-36) Taiwan version. SF-36 is composed of 36 items, which form two summary scales, namely the physical component score (PCS) and the mental component score (MCS). Higher scores on the PCS and MCS indicate a better physical and mental aspect of HRQOL, respectively [48,49]. The reliability and validity of the SF-36 Taiwan version have been well-established [50,51].

The secondary outcomes in this study were BMI, dietary behavior, and PA. Dietary behavior was assessed by the dietary behavior questionnaire, which consisted of 14 items based on a 4-point Likert scale, ranging from 1 (never) to 4 (always). The scores ranged from 14 to 56, with higher scores indicating a better correspondence to the DASH diet [42]. PA was assessed by the International Physical Activity Questionnaire Taiwan version, which contained 7 questions about the frequency, duration, and intensity of PA in the last 7 days. The results could be further classified into mild, moderate, and vigorous PA and quantified into metabolic equivalents (MET), expressed as MET-minutes/week [52].

### Statistical Analysis

The statistical analyses were performed using IBM SPSS Statistics for Windows, version 23.0 (IBM Corp, Armonk, NY, USA). We analyzed the data using an intention-to-treat analysis. For participants with incomplete or missing data, we used the maximum-likelihood method for imputation [53].

Descriptive characteristics are presented as percentages or as means and SD, as appropriate. We used the paired *t*-test to examine the changes in outcome variables within groups. One-way ANOVA was used for comparisons among groups with the Scheffe posthoc test for pairwise comparisons. Finally, we used generalized estimating equations (GEEs) to estimate the intervention effects after adjusting for age, gender, and baseline value of the outcome variables.

## Results

### Participant Characteristics

The characteristics of the three groups are shown in Table 1. The mean age of the participants was 58.08 (SD 10.21) years (range 29-86 years). Approximately 90% of participants reported a history of chronic disease. There were no significant differences in sociodemographics, disease history, and cigarette and alcohol use among the three groups.

**Table 1:** Characteristics of the participants in the three groups.

Characteristics		Total (N=121)	CG^a^ (n=38)	OMG^b^ (n=41)	TCMG^c^ (n=42)	*χ*^2^ or *F*^d^ (*df*)	*P* value
Age (years), mean (SD)		58.08 (10.21)	60.14 (10.83)	56.93 (10.88)	57.34 (8.81)	1.15 (2)	.32
**Sex, n (%)**						2.11 (2)	.35
	Female	64 (52.9)	18 (47.4)	20 (48.8)	26 (61.9)		
	Male	57 (47.1)	20 (52.6)	21 (51.2)	16 (38.1)		
**Currently married, n (%)**						1.69 (2)	.43
	No	28 (23.1)	6 (15.8)	11 (26.8)	11 (26.2)		
	Yes	93 (76.9)	32 (84.2)	30 (73.2)	31 (73.8)		
**Education level, n (%)**						3.61 (4)	.46
	Elementary school or below	10 (8.3)	5 (13.2)	2 (4.9)	3 (7.1)		
	Junior and senior high school	48 (39.7)	12 (31.6)	16 (39.0)	20 (47.6)		
	University or above	63 (52.1)	21 (55.3)	23 (56.1)	19 (45.2)		
**Employment status, n (%)**						1.18 (2)	.55
	Unemployed	49 (40.5)	18 (47.4)	16 (39.0)	15 (35.7)		
	Employed	72 (59.5)	20 (52.6)	25 (61.0)	27 (64.3)		
**History of chronic disease, n (%)**						1.03 (2)	.60
	No	13 (10.7)	3 (7.9)	6 (14.6)	4 (9.5)		
	Yes	108 (89.3)	35 (92.1)	35 (85.4)	38 (90.5)		
**Smoking status, n (%)**						6.81 (4)	.15
	Never	97 (80.2)	29 (76.3)	30 (73.2)	38 (90.5)		
	Quit	20 (16.5)	8 (21.1)	10 (24.4)	2 (4.8)		
	Current smoker	4 (3.3)	1 (2.6)	1 (2.4)	2 (4.8)		
**Alcohol drinking, n (%)**						5.82 (4)	.21
	Never	106 (87.6)	32 (84.2)	35 (85.4)	39 (92.9)		
	Quit	4 (3.3)	2 (5.3)	0 (0.0)	2 (4.8)		
	Current drinker	11 (9.1)	4 (10.5)	6 (14.6)	1 (2.4)		

^a^CG: control group.

^b^OMG: ordinary mobile health app group.

^c^TCMG: traditional Chinese medicine mobile health app group.

^d^*F* and *χ*^2^ are the respective values of one-way ANOVA and Pearson *χ^2^* test.

### Overall Intervention Effects

**Figure 2:**
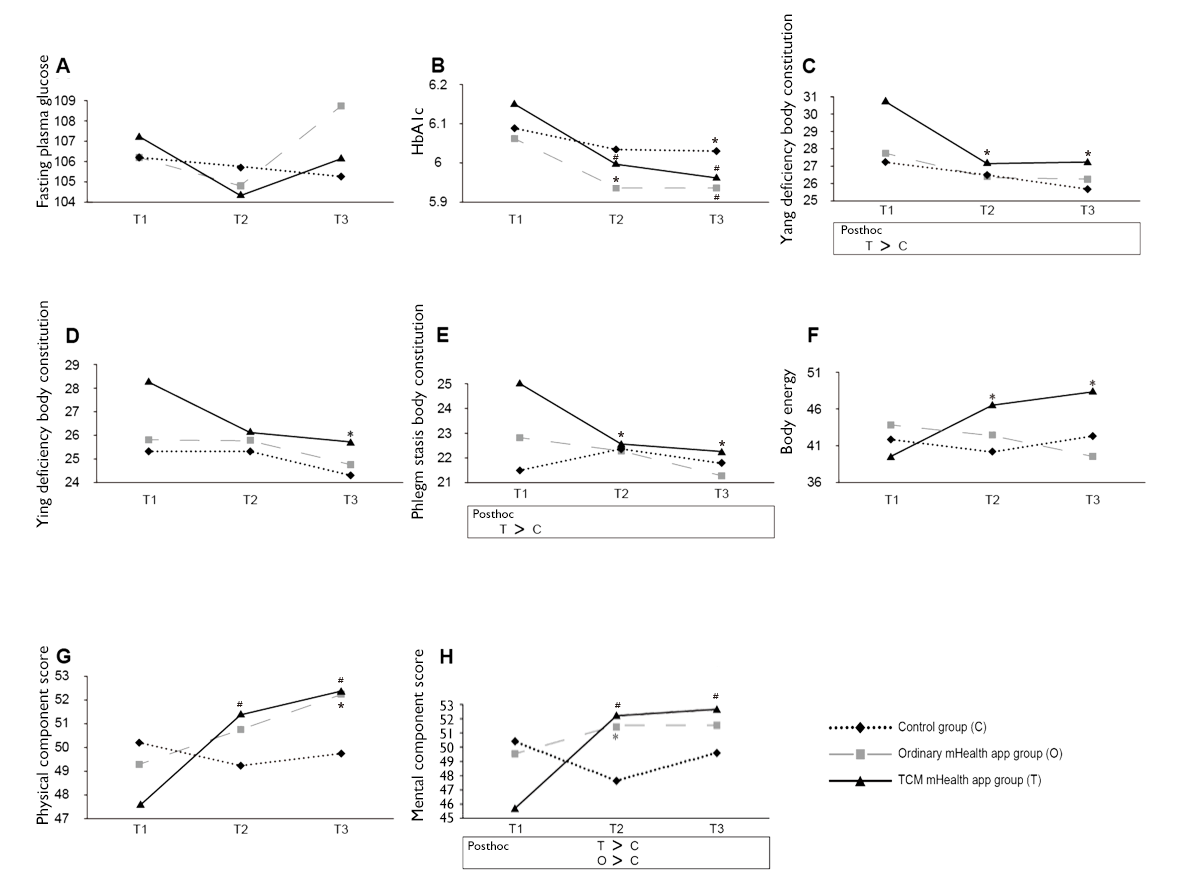
Changes in primary outcomes. (A) Fasting plasma glucose. (B) HbA_1c_. (C) Yang deficiency body constitution. (D) Ying deficiency body constitution. (E) Phlegm stasis body constitution. (F) Body energy. (G) Physical component score. (H) Mental component score. Within-group across-time comparisons were made from a paired *t* test with T1 as the reference. Between-group comparisons were based on one-way ANOVA with the Scheffe posthoc test, with the results presented below graphs. mHealth: mobile health; T1: baseline; T2: end of the intervention; T3: 1 month after the intervention; TCM: traditional Chinese medicine. *P<.05, #*P*<.001.

**Figure 3:**
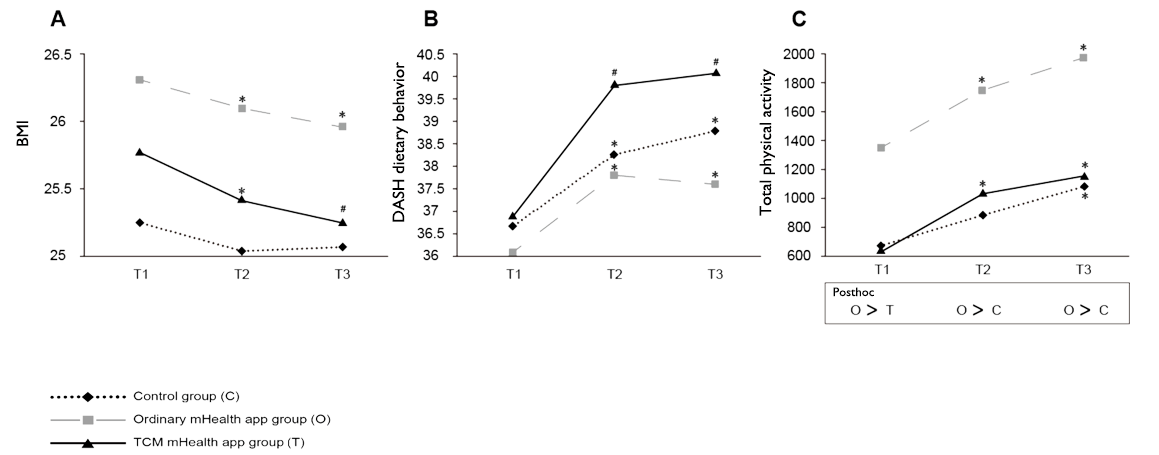
Changes in secondary outcomes. (A) BMI. (B) DASH dietary behavior. (C) Total physical activity. Within-group across-time comparisons were made from a paired *t* test with T1 as the reference. Between-group comparisons were based on one-way ANOVA with the Scheffe posthoc test, with the results presented below the graph in (C). mHealth: mobile health; T1: baseline; T2: end of the intervention; T3: 1 month after the intervention; TCM: traditional Chinese medicine. *P<.05, #*P*<.001.

The crude effects of the intervention on the outcomes are shown in Figures 2 and 3 (details are shown in Multimedia Appendix 5). Of the outcomes included, there were significant differences in *yang-*deficiency and phlegm-stasis body constitution among the three groups, with the TCM mHealth app group scoring higher than the control group (Figure 2). In addition, the ordinary mHealth app group reported the highest amount of PA among the three groups (Figure 3). For body constitution and PA, GEE results are preferred over the crude results given baseline differences. There were no significant differences in other outcomes between the groups at preintervention. Net effects of the intervention on the outcomes are shown in Table 2 (full model results are shown in Multimedia Appendix 6) using the control group as the reference. To explicitly compare the effects between the TCM and ordinary mHealth groups, Table 3 shows the results using the ordinary mHealth group as the reference (full model results are shown in Multimedia Appendix 7).

**Table 2:** Generalized estimating equation models to compare the differences among the three groups, using the control group as the reference.^a^

Parameter	OMG^b^×T2^c^, β (95% CI)	OMG×T3^d^, β (95% CI)	TCMG^e^×T2, β (95% CI)	TCMG×T3, β (95% CI)
FPG^f^	−1.18 (−5.92 to 3.56)	3.17 (−2.22 to 8.56)	−2.52 (−7.39 to 2.35)	−.37 (−5.61 to 4.86)
HbA_1c_^g^	−.06 (−.15 to .04)	−.05 (−.14 to .04)	−.08 (−.18 to .02)	−.11 (−.21 to −.01)
Yang-deficiency BC^h^	–.81 (−3.16 to 1.53)	−.46 (−2.73 to 1.81)	−3.15 (−6.09 to −.21)	−2.37 (−5.04 to .29)
Yin-deficiency BC	–.02 (–2.56 to 2.52)	.03 (–2.08 to 2.13)	–2.29 (–5.28 to .70)	–1.62 (–4.21 to .96)
Phlegm-statis BC	−1.36 (−4.16 to 1.44)	−1.88 (−4.38 to .63)	−3.45 (−6.49 to −.42)	−3.30 (−6.01 to −.58)
Body energy	–.16 (−11.23 to 10.91)	−5.84 (−17.68 to 6.01)	8.60 (−1.91 to 19.11)	7.81 (−3.36 to 18.98)
PCS^i^	2.56 (−.44 to 5.56)	2.90 (−.07 to 5.89)	4.93 (1.97 to 7.89)	4.89 (1.92 to 7.87)
MCS^j^	4.63 (.91 to 8.35)	2.68 (−1.16 to 6.51)	8.11 (3.82 to 12.40)	7.26 (3.35 to 11.17)
BMI	−.03 (−.34 to .29)	−.24 (−.61 to .13)	−.12 (−.43 to .20)	−.37 (−.73 to −.02)
DASH^k^ dietary behavior	.22 (−1.42 to 1.86)	−.51 (−2.49 to 1.48)	1.21 (−.62 to 3.06)	.99 (−1.05 to 3.04)
Total physical activity	236.33 (−231.76 to 704.43)	213.18 (−360.50 to 786.86)	248.59 (−191.18 to 688.37)	122.74 (−459.22 to 704.71)

^a^Interaction effects were examined after adjustments for age and gender, and the baseline value of the outcome variable; baseline and control group served as references.

^b^OMG: ordinary mobile health app group.

^c^T2: end of the intervention.

^d^T3: 1 month after the intervention.

^e^TCMG: traditional Chinese medicine mobile health app group.

^f^FPG: fasting plasma glucose.

^g^HbA_1c_: hemoglobin A_1c_.

^h^BC: body constitution.

^i^PCS: physical component score.

^j^MCS: mental component score.

^k^DASH: Dietary Approaches to Stop Hypertension.

**Table 3:** Generalized estimating equation models to compare the outcomes between the TCM mHealth app group (n=42) and ordinary mHealth app group (n=41).^a^

Parameter	TCMG^b^×T2^c^, β (95% CI)	TCMG×T3^d^, β (95% CI)
FPG^e^	−1.82 (−6.30 to 2.66)	3.96 (−9.91 to 1.99)
HbA_1c_^f^	−.02 (−.12 to .09)	−.06 (−.15 to .04)
Yang- deficiency BC^g^	−2.26 (−5.12 to .61)	−1.96 (−4.83 to .92)
Yin-deficiency BC	−2.16 (–5.00 to .68)	−1.40 (–4.20 to 1.41)
Phlegm-statis BC	−.24 (−5.19 to .70)	−1.55 (−4.03 to .94)
Body energy	8.65 (−2.11 to 19.41)	12.30 (1.31 to 23.30)
PCS^h^	2.32 (−.58 to 5.22)	2.24 (−.87 to 5.35)
MCS^i^	3.14 (−.82 to 7.10)	4.29 (.27 to 8.31)
BMI	−.10 (−.41 to .21)	−.14 (−.49 to .20)
DASH^j^ dietary behavior	1.06 (−.48 to 2.60)	1.44 (−.45 to 3.33)
Total physical activity	4.99 (−535.31 to 545.30)	−82.67 (−708.14 to 542.80)

^a^Interaction effects were examined after adjustments for age and gender and the baseline value of the outcome variable; baseline and ordinary mobile health group served as references.

^b^TCMG: traditional Chinese medicine mobile health app group.

^c^T2: end of the intervention.

^d^T3: 1 month after the intervention.

^e^FPG: fasting plasma glucose.

^f^HbA_1c_: hemoglobin A_1c_.

^g^BC: body constitution.

^h^PCS: physical component score.

^i^MCS: mental component score.

^j^DASH: Dietary Approaches to Stop Hypertension.

### Primary Outcomes

#### Blood Sugar Control

There were no significant differences in the FPG and HbA_1c_ among the three groups at the three time points. FPG did not change significantly at postintervention and 1 month after the intervention as compared to the preintervention (baseline) levels for all three groups. However, HbA_1c_ decreased significantly over time for all three groups (Figure 2; Multimedia Appendix 5).

The GEE analyses revealed no significant group differences in FPG. The HbA_1c_ levels decreased significantly in the TCM mHealth app group at T3 and were significantly different than those in control group, but there were no significant differences between the ordinary mHealth app and control groups (Table 2; Multimedia Appendix 6).

#### Body Constitution

The TCM mHealth app group scored the highest in *yang*-deficiency and phlegm-stasis body constitution at T1, with significant differences between the TCM and control groups. There were no significant differences in body constitution of the three groups at T2 and T3. All three types of body constitutions improved significantly at T3 for the TCM group, but no such improvements were observed in the ordinary mHealth app and control groups (Figure 2; Multimedia Appendix 5).

The GEE analyses indicated that the TCM mHealth app group showed significant improvements in *yang-*deficiency body constitution at T2 compared to that in the control group. Furthermore, greater improvement in phlegm-stasis body constitution was found compared to that in the control group, and the effect persisted until 1 month after the intervention. However, no effect on *yin-*deficiency body constitution was observed (Table 2; Multimedia Appendix 6). There were no significant differences in body constitution between the TCM and ordinary mHealth app groups (Table 3; Multimedia Appendix 7).

#### Body Energy

There were no significant differences in body energy among the three groups at the three time points. Body energy increased significantly from T1 to T2 and from T1 to T3 in the TCM mHealth app group, but remained unchanged in the ordinary mHealth app and control groups (Figure 2; Multimedia Appendix 5).

The GEE results indicated that the TCM mHealth app group showed a significant increase in body energy at T3 compared to that in the ordinary mHealth group (Table 3; Multimedia Appendix 7).

#### Health-Related Quality of Life

There were no significant differences in the PCS among the three groups at the three time points. The TCM mHealth app group had a significant increase in the PCS from T1 to T2 and from T1 to T3. The ordinary mHealth app group also had a significant increase in the PCS from T1 to T3 (Figure 2; Multimedia Appendix 5).

With regard to the MCS, the TCM mHealth app group had a significantly higher score at T2 compared with that of the control group (*P*=.02), as did the ordinary mHealth app group (*P*=.03; Figure 2; Multimedia Appendix 5). The TCM mHealth app group showed a significant increase in the MCS from T1 to T2 and from T1 to T3. Moreover, the ordinary mHealth app group had a significant increase in the MCS from T1 to T2 and from T1 to T3 (with borderline significance; *P*=.04 and *P*=.05, respectively).

The GEE results indicated that the TCM mHealth app group had a significant increase in the PCS and MCS at T2 and T3 compared with that of the control group (Table 2; Multimedia Appendix 6). In addition, the increase in the MCS in the TCM mHealth app group from T1 to T3 appeared to be higher than that in the ordinary mHealth app group (Table 3; Multimedia Appendix 7). The ordinary mHealth app group had a significant increase in the MCS at T2 compared to that of the control group (Table 2; Multimedia Appendix 6).

### Secondary Outcomes

#### BMI

There were no significant differences in BMI among the three groups at the three time points (Figure 3; Multimedia Appendix 5). The BMI in the TCM and ordinary mHealth app groups decreased significantly from T1 to T2 and from T1 to T3. However, the BMI of the control group remained unchanged.

The GEE results indicated that the TCM mHealth app group showed a significant decrease in BMI at T3 compared to that in the control group (Table 2; Multimedia Appendix 6). However, there were no significant differences in the change in BMI between the TCM and ordinary mHealth app groups (Table 3; Multimedia Appendix 7).

#### Dietary Behavior

There were no significant differences in the DASH dietary behavior among the three groups at the three time points (Figure 3; Multimedia Appendix 5). The DASH dietary behavior improved significantly in all three groups from T1 to T2 and from T1 to T3. The GEE results showed no significant differences in the DASH dietary behavior among the three groups over time (Tables 2 and 3; Multimedia Appendices 6 and 7).

#### Physical Activity

The ordinary mHealth app group had a significantly higher PA level than that of the other two groups across all three time points (Figure 3; Multimedia Appendix 5). The PA increased significantly from T1 to T3 in all three groups. The GEE results showed no significant differences in the total PA among the three groups over time (Tables 2 and 3; Multimedia Appendices 6 and 7).

## Discussion

### Principal Findings

This RCT found that the TCM mHealth group showed better HbA_1c_, *yang*-deficiency body constitution, phlegm-stasis body constitution, physical aspect of HRQOL, mental aspect of HRQOL, and BMI than the control group. When the TCM and ordinary mHealth app groups were compared, the TCM mHealth app group showed higher body energy and mental aspect scores of the HRQOL at 1 month after intervention than the ordinary mHealth app group. These results suggest that the TCM mHealth app helps to effectively control blood sugar, decrease *yang-*deficiency and phlegm-stasis body constitution, and improve body energy and HRQOL. Incorporating TCM body constitution and meridian energy concepts into an mHealth app seems plausible and can improve health for individuals with prediabetes. According to TCM theory, an improved body constitution could decrease susceptibility to chronic diseases [14] and increased body energy would manifest in better stamina [17]. Therefore, improved body constitution and increased body energy could explain the effectiveness of the TCM mHealth app for improving HbA_1c_ and HRQOL. Further studies are needed to examine the interrelationships among these outcomes.

Previous studies showed that using an mHealth app improved HbA_1c_, dietary behavior, PA, and BMI compared to those of controls [10-12]. However, we did not find such an effect for the ordinary mHealth app group when compared with the control group in this study. It was noted that all participants in the study had received 15-20 minutes of health education about the disease, healthy diet, and exercise encouragement when they were diagnosed with prediabetes in the study hospital. Possibly owing to this health education, we found that the HbA_1c_, DASH dietary behavior, and PA improved over time for all three groups. Such universal improvement in health behavior may be the reason for the lack of significant effect on these outcomes when the ordinary mHealth app group was compared to the controls.

The TCM dietary and PA advice used in the TCM mHealth app is mainly based on the types of foods/PA to avoid and those to consume/practice based on the individual’s body constitution. Qigong, including belly breathing and Baduanjin, was recommended for all participants since these exercises can improve *qi* and are appropriate for all types of body constitutions. The recommended types of foods consumed differed according to the individual’s body constitution. We found that the TCM dietary and PA principles do not conflict with the principles of the DASH diet and recommended PA amount, except that individuals with a *yin*-deficiency body constitution were advised to avoid vigorous PA.

When compared to the ordinary mHealth app group, the TCM mHealth app group did not differ significantly in improving body constitution and HbA_1c_. However, the TCM mHealth app group showed better improvement in the mental aspect of HRQOL and in increasing body energy than the ordinary mHealth app group. These results imply that using the TCM mHealth app can better improve body energy and HRQOL in individuals with prediabetes compared to the ordinary mHealth app. This may be because people who practice TCM consider that health conditions can improve with sufficient *qi*. The importance of these indicators across cultures needs to be examined in future studies.

Our study showed that both the TCM and ordinary mHealth app groups exhibited a significant improvement in HbA_1c_ and HRQOL with time. This finding is consistent with a previous meta-analysis that reported a positive effect of a lifestyle intervention on HbA_1c_ in individuals with prediabetes [54]. Another meta-analysis reported positive effects of Qigong in improving the HRQOL [55]. Another study demonstrated that individuals with prediabetes who achieved moderate-intensity PA (≥150 minutes/week) have higher levels of HRQOL than inactive people [56]. Therefore, the mHealth app can be used to assist individuals with prediabetes to increase their PA and HRQOL, while decreasing their HbA_1c_.

This study showed effectiveness of the intervention in HbA_1c_ but not FPG. The insignificant FPG results may be explained as follows. First, various factors can influence FPG, such as emotional state, drugs, and stress hormones [57]. Thus, FPG is not a stable estimate of glycemic exposure [58]. Second, although we encouraged the participants to fast for at least 8 hours, we are unsure if they followed these instructions. In future studies, we suggest that more than two tests should be used to enhance diagnostic accuracy.

In this study, we found that the TCM mHealth app effectively improved the *yang-*deficiency and phlegm-stasis body constitution in individuals with prediabetes, but could not improve the *yin*-deficiency body constitution. More research is needed to develop effective interventions to improve the *yin*-deficiency body constitution among individuals with prediabetes.

Finally, the TCM mHealth app group did show significant improvements in body constitution and body energy in our study, while the other two groups did not show changes in these factors over time. These results suggest that medical practitioners could provide the TCM mHealth app to individuals with prediabetes, through which Qigong and a Chinese dietary regimen can improve body energy, body constitution, and HRQOL. Studies with a larger sample size and longer follow-up period are needed to compare the effectiveness of the two different approaches.

### Limitations

This study has several limitations. First, most participants were from an outpatient department and had chronic conditions. Therefore, this sample may have had more complex health problems than present in people with prediabetes alone. The participants may be more homogeneous since they were from one single center. Second, the sample size was small. The limited sample size may be the reason for some statistically insignificant results when comparing between groups. Third, the study was an open-label trial where participants were aware of their group assignments; thus, performance bias was possible. Fourth, the study used a 12-week intervention and a 1-month follow-up period according to previous TCM lifestyle programs [35,37]. A follow-up period from 12 to 24 months may be preferred in future studies. Fifth, the use of the mHealth apps in the follow-up period was not monitored. In addition, during the intervention period, the participants were reminded to use the apps, but the time spent on app use varied, suggesting that the participation level may be different. Lastly, this study included three types of body constitutions. There are many other types of body constitutions that could be considered in future studies.

### Conclusion

We developed a TCM mHealth app to incorporate TCM concepts into an mHealth app for individuals with prediabetes. Compared to controls not using the app, the TCM mHealth app appeared to be effective in improving HbA_1c_, BMI, *yang*-deficiency and phlegm-stasis body constitution, and HRQOL. Compared to individuals using the ordinary mHealth app, individuals using the TCM mHealth app showed higher body energy and mental aspects of the HRQOL 1 month after the intervention. The TCM mHealth app was not effective in improving FPG, *yin*-deficiency body constitution, DASH dietary behavior, and total PA. The study results suggest that individuals with prediabetes could use the TCM mHealth app to improve their body energy and HRQOL. Further studies with a larger sample size and a longer follow-up period are warranted to verify whether the differences favoring the TCM app are clinically meaningful.

## Appendix

Multimedia Appendix 1 Definitions of terms in traditional Chinese medicine.

Multimedia Appendix 2 Screenshots of the ordinary and TCM mHealth apps.

Multimedia Appendix 3 Taxonomy of the behavior change techniques used in the mHealth app.

Multimedia Appendix 4 CONSORT-EHEALTH checklist.

Multimedia Appendix 5 Comparison of the primary and secondary outcomes among the TCM mHealth app, ordinary mHealth app, and control groups (N=121).

Multimedia Appendix 6 Generalized estimating equation models to compare the differences among the three groups, using the control group as the reference.

Multimedia Appendix 7 Generalized estimating equation models to compare the outcomes between the TCM mHealth app (n=42) and ordinary mHealth app groups (n=41).

## Abbreviations

BCQ: Body Constitution Questionnaire
DASH: Dietary Approaches to Stop Hypertension
DPP: Diabetes Prevention Program
FPG: fasting plasma glucose
GEE: generalized estimating equation
HbA_1c_: hemoglobin A_1c_
HRQOL: health-related quality of life
MCS: mental component score
MEAD: Meridian Energy Analysis Device
MET: metabolic equivalent
mHealth: mobile health
PA: physical activity
PCS: physical component score
RCT: randomized controlled trial
SF-36: Medical Outcome Survey Short-Form
T2DM: type 2 diabetes mellitus
TCM: traditional Chinese medicine

## Data Availability

All data generated or analyzed during this study are included in this published article and its supplementary information files.

## References

[1] Song Y, Wang H, Qin L, Li M, Gao S, Wu L, Liu T (2020). Efficiency and safety of Chinese herbal medicine in the treatment of prediabetes: a systemic review and meta-analysis of randomized controlled trials. Evid Based Complement Alternat Med.

[2] American Diabetes Association (2014). Diagnosis and classification of diabetes mellitus. Diabetes Care.

[3] National diabetes statistics report 2020: Estimates of diabetes and its burden in the United States. Centers for Disease Control and Prevention.

[4] Bansal N (2015). Prediabetes diagnosis and treatment: A review. World J Diabetes.

[5] Knowler WC, Barrett-Connor E, Fowler SE, Hamman RF, Lachin JM, Walker EA, Nathan DM, Diabetes Prevention Program Research Group (2002). Reduction in the incidence of type 2 diabetes with lifestyle intervention or metformin. N Engl J Med.

[6] Ely EK, Gruss SM, Luman ET, Gregg EW, Ali MK, Nhim K, Rolka DB, Albright AL (2017). A national effort to prevent type 2 diabetes: participant-level evaluation of CDC's National Diabetes Prevention Program. Diabetes Care.

[7] Mensa-Wilmot Y, Bowen S, Rutledge S, Morgan JM, Bonner T, Farris K, Blacher R, Rutledge G (2017). Early results of states' efforts to support, scale, and sustain the National Diabetes Prevention Program. Prev Chronic Dis.

[8] Atienza AA, Patrick K (2011). Mobile health: the killer app for cyberinfrastructure and consumer health. Am J Prev Med.

[9] Joiner KL, Nam S, Whittemore R (2017). Lifestyle interventions based on the diabetes prevention program delivered via eHealth: a systematic review and meta-analysis. Prev Med.

[10] Toro-Ramos T, Michaelides A, Anton M, Karim Z, Kang-Oh L, Argyrou C, Loukaidou E, Charitou MM, Sze W, Miller JD (2020). Mobile delivery of the Diabetes Prevention Program in people with prediabetes: randomized controlled trial. JMIR Mhealth Uhealth.

[11] Block G, Azar KM, Romanelli RJ, Block TJ, Hopkins D, Carpenter HA, Dolginsky MS, Hudes ML, Palaniappan LP, Block CH (2015). Diabetes prevention and weight loss with a fully automated behavioral intervention by email, web, and mobile phone: a randomized controlled trial among persons with prediabetes. J Med Internet Res.

[12] Block G, Azar KMJ, Romanelli RJ, Block TJ, Palaniappan LP, Dolginsky M, Block CH (2016). Improving diet, activity and wellness in adults at risk of diabetes: randomized controlled trial. Nutr Diabetes.

[13] Sarrafzadegan N, Kelishadi R, Esmaillzadeh A, Mohammadifard N, Rabiei K, Roohafza H, Azadbakht L, Bahonar A, Sadri G, Amani A, Heidari S, Malekafzali H (2009). Do lifestyle interventions work in developing countries? Findings from the Isfahan Healthy Heart Program in the Islamic Republic of Iran. Bull World Health Organ.

[14] Huang Y, Lin C, Cheng S, Lin C, Lin SJ, Su Y (2019). Using Chinese body constitution concepts and measurable variables for assessing risk of coronary artery disease. Evid Based Complement Alternat Med.

[15] Wang Q (2012). Individualized medicine, health medicine, and constitutional theory in Chinese medicine. Front Med.

[16] Huang S, Chien L, Chang C, Chen P, Tai C (2011). Abnormal gastroscopy findings were related to lower meridian energy. Evid Based Complement Alternat Med.

[17] Bao GC (2020). The idealist and pragmatist view of qi in tai chi and qigong: a narrative commentary and review. J Integr Med.

[18] Wang G, Ayati MH, Zhang W (2010). Meridian studies in China: a systematic review. J Acupunct Meridian Stud.

[19] Huang S, Chien L, Tai C, Chen P, Lien P, Tai C (2015). Effects of symptoms and complementary and alternative medicine use on the yang deficiency pattern among breast cancer patients receiving chemotherapy. Complement Ther Med.

[20] Lin J, Chen L, Lin J, Chang C, Huang Y, Su Y (2012). BCQ-: a body constitution questionnaire to assess Yin-Xu. Part I: establishment of a provisional version through a Delphi process. Forsch Komplementmed.

[21] Su Y, Chen L, Lin J, Lin J, Huang Y, Lai J (2008). BCQ+: a body constitution questionnaire to assess Yang-Xu. Part I: establishment of a first final version through a Delphi process. Forsch Komplementmed.

[22] Lin J, Lin J, Chen L, Chang C, Huang Y, Su Y (2012). BCQs: a Body Constitution Questionnaire to assess stasis in traditional Chinese medicine. Eur J Integr Med.

[23] Wang Q (2005). Classification and diagnosis basis of nine basic constitutions in Chinese medicine. J Beijing Univ Trad Chin Med.

[24] Tsai C, Su Y, Lin S, Lee I, Lee C, Li T (2014). Reduced health-related quality of life in body constitutions of yin-xu, and yang-xu, stasis in patients with type 2 diabetes: taichung diabetic body constitution study. Evid Based Complement Alternat Med.

[25] Kung Y, Kuo TBJ, Lai C, Shen Y, Su Y, Yang CCH (2021). Disclosure of suboptimal health status through traditional Chinese medicine-based body constitution and pulse patterns. Complement Ther Med.

[26] Chung H, Chien L, Huang S, Tai C, Tai C (2016). Changes in symptom patterns and health-related quality of life of cancer patients before and after chemotherapy. J Tradit Chin Med.

[27] Li Lingru, Yao Haiqiang, Wang Ji, Li Yingshuai, Wang Qi (2019). The Role of Chinese Medicine in Health Maintenance and Disease Prevention: Application of Constitution Theory. Am J Chin Med.

[28] Sancier Kenneth M (2003). Electrodermal measurements for monitoring the effects of a qigong workshop. J Altern Complement Med.

[29] Weng Ching-Sung, Hung Yu-Li, Shyu Liang-Yu, Chang Yung-Hsien (2004). A study of electrical conductance of meridian in the obese during weight reduction. Am J Chin Med.

[30] (2000). Measuring healthy days: Population assessment of health-related quality of life. Centers for Disease Control and Prevention,.

[31] Ware J E, Gandek B (1998). Overview of the SF-36 Health Survey and the International Quality of Life Assessment (IQOLA) Project. J Clin Epidemiol.

[32] Ji J, Yang S, Lou Q, Liu C (2018). Effect of traditional Chinese medicine meridian massage on prediabetes patients. J Hunan Univ Chin Med.

[33] Wang Y, Wu X, Wang HHX, Li Y, Fu Y, Wang J, Hernandez J, Wong MCS (2021). Body constitution and unhealthy lifestyles in a primary care population at high cardiovascular risk: new insights for health management. Int J Gen Med.

[34] Sun H, Tian W, Wu S, Chang L, Lung H, Liu C (2016). Traditional Chinese medicine non-drug comprehensive program in the treatment of impaired glucose tolerance: a clinical observation. Hebei J Trad Chin Med. in Chinese.

[35] Liou X, Wang C, Liu Z, Li Y, Chen F, Wu Y (2016). Study on effect of TCM health education on the behavior changes of prediabetic state patients under internet mode. J Chin Med.

[36] Liu X, Miller YD, Burton NW, Brown WJ (2010). A preliminary study of the effects of Tai Chi and Qigong medical exercise on indicators of metabolic syndrome, glycaemic control, health-related quality of life, and psychological health in adults with elevated blood glucose. Br J Sports Med.

[37] Geng BC, Wu Y, Jin ZF, Dang YY (2017). Effect of health education on TCM blood glucose index and behavior in patients with diabetes mellitus under internet mode. J Clin Nurs.

[38] Wang Y, Zhu YH, Zhang XT, Cheng F (2017). Clinical observation of comprehensive nondrug interventions of traditional Chinese medicine in community prediabetes population. Shanghai J Trad Chin Med.

[39] Staite E, Bayley A, Al-Ozairi E, Stewart K, Hopkins D, Rundle J, Basudev N, Mohamedali Z, Ismail K (2020). A wearable technology delivering a web-based diabetes prevention program to people at high risk of type 2 diabetes: randomized controlled trial. JMIR Mhealth Uhealth.

[40] Ibrahim N, Ming Moy F, Awalludin IAN, Mohd Ali Z, Ismail IS (2016). Effects of a community-based healthy lifestyle intervention program (Co-HELP) among adults with prediabetes in a developing country: a quasi-experimental study. PLoS One.

[41] Pengpid S, Peltzer K, Skaal L (2014). Efficacy of a church-based lifestyle intervention programme to control high normal blood pressure and/or high normal blood glucose in church members: a randomized controlled trial in Pretoria, South Africa. BMC Public Health.

[42] Yang L, Lin C, Lin T, Liu T (2013). Effects of a self-regulation program on dietary behavior, physical activity, and physiological measures among prediabetic adults: a pilot study. J Nurs Healthc Res.

[43] Michie S, Richardson M, Johnston M, Abraham C, Francis J, Hardeman W, Eccles MP, Cane J, Wood CE (2013). The behavior change technique taxonomy (v1) of 93 hierarchically clustered techniques: building an international consensus for the reporting of behavior change interventions. Ann Behav Med.

[44] Eysenbach G, CONSORT-EHEALTH Group (2011). CONSORT-EHEALTH: improving and standardizing evaluation reports of Web-based and mobile health interventions. J Med Internet Res.

[45] Wong W, Lam CLK, Su Y, Lin SJ, Ziea ET, Wong VT, Wai LK, Kwan AKL (2014). Measuring body constitution: validation of the Body Constitution Questionnaire (BCQ) in Hong Kong. Complement Ther Med.

[46] Chang S, Weng Y, Cheng S, Chang Y, Lee T, Chang C, Chang T, Huang K, Liu C, Hsu C (2019). Application of meridian electrical conductance in the setting of acute ischemic stroke: a cross-sectional study. Evid Based Complement Alternat Med.

[47] Lee Y, Ng HP, Chang Y, Ho W (2018). The development and application evaluation of meridian energy detection system in traditional Oriental medicine: a preliminary study. Evid Based Complement Alternat Med.

[48] McHorney CA, Ware JE, Lu JF, Sherbourne CD (1994). The MOS 36-item Short-Form Health Survey (SF-36): III. Tests of data quality, scaling assumptions, and reliability across diverse patient groups. Med Care.

[49] McHorney CA, Ware JE, Raczek AE (1993). The MOS 36-Item Short-Form Health Survey (SF-36): II. Psychometric and clinical tests of validity in measuring physical and mental health constructs. Med Care.

[50] Fuh JL, Wang SJ, Lu SR, Juang KD, Lee SJ (2000). Psychometric evaluation of a Chinese (Taiwanese) version of the SF-36 health survey amongst middle-aged women from a rural community. Qual Life Res.

[51] Tseng H, Lu JR, Gandek B (2003). Cultural issues in using the SF-36 Health Survey in Asia: results from Taiwan. Health Qual Life Outcomes.

[52] Bergier J, Kapka-Skrzypczak L, Biliński P, Paprzycki P, Wojtyła A (2012). Physical activity of Polish adolescents and young adults according to IPAQ: a population based study. Ann Agric Environ Med.

[53] Ibrahim JG, Molenberghs G (2009). Missing data methods in longitudinal studies: a review. Test (Madr).

[54] Sun Y, You W, Almeida F, Estabrooks P, Davy B (2017). The effectiveness and cost of lifestyle interventions including nutrition education for diabetes prevention: a systematic review and meta-analysis. J Acad Nutr Diet.

[55] Wang X, Pi Y, Chen B, Chen P, Liu Y, Wang R, Li X, Zhu Y, Yang Y, Niu Z (2015). Effect of traditional Chinese exercise on the quality of life and depression for chronic diseases: a meta-analysis of randomised trials. Sci Rep.

[56] Taylor LM, Spence JC, Raine K, Plotnikoff RC, Vallance JK, Sharma AM (2010). Physical activity and health-related quality of life in individuals with prediabetes. Diabetes Res Clin Pract.

[57] Kyrou I, Tsigos C (2009). Stress hormones: physiological stress and regulation of metabolism. Curr Opin Pharmacol.

[58] Kaur G, Lakshmi PVM, Rastogi A, Bhansali A, Jain S, Teerawattananon Y, Bano H, Prinja S (2020). Diagnostic accuracy of tests for type 2 diabetes and prediabetes: a systematic review and meta-analysis. PLoS One.

