# Supplementary material for: The Effectiveness of a Traditional Chinese Medicine–Based Mobile Health App for Individuals With Prediabetes: Randomized Controlled Trial
Source: JMIR Mhealth Uhealth. 2023 Jun 20;11:e41099. doi: 10.2196/41099 (PMC10337399; doi:10.2196/41099)
Supplement: Multimedia Appendix 1 [file mhealth_v11i1e41099_app1.pdf]

Multimedia Appendix 1. Definitions of words

Words	Definitions
Body constitution	Body constitution is the individual's body condition that makes an individual susceptible to certain diseases but not others [14]. It refers to an integrated, metastable, and natural tendency of an individual, which may manifest itself in morphological structures, physiological functions, and psychological status. A person's body constitution is affected by congenital as well as acquired factors, such as disease, diet, and physical activity [27]. Types of body constitutions can be classified into *yin*-deficiency, *yang*-deficiency, and phlegm-stasis [20-22, 26]. *Yang*-deficiency refers to insufficiency of *qi* (energy). Individuals with this deficiency may experience symptoms such as fatigue, shortness of breath, chills, loose stool, and a large volume of urine. *Yin*-deficiency reflects an insufficiency in blood and interstitial fluids, and thus, patients with *yin*- deficiency may experience symptoms such as being constantly thirsty, experiencing hot flushes, hard stool, and a low volume of urine. Phlegm is a viscous and turbid pathological factor that is formed due to an imbalance in body fluid. Phlegm-stasis refers to the accumulation of phlegm in the body as a form of condensation, which results in dizziness, chest tightness, and numbness in the limbs. The classification is important to help TCM practitioners to develop individualized treatment plans and preventive regimens. Therefore, the body constitution may be modified to strengthen the body constitution and thus become less susceptible to disease. Traditional Chinese medicine (TCM) concepts of blood, phlegm, and fluids are not equivalent to the Western uses of these terms, but rather are used to present energetic qualities. In TCM, blood is considered a vehicle for *qi* and that carries inherent energy. It nourishes and moistens the body, and it circulates nutritive *qi*. According to TCM theory, body fluids originate from food and water. They are formed during digestion. Fluids refer to different kinds of physiological fluids, including fluids in the organs and tissues, gastric fluid, intestinal fluid, semen, and tears. Phlegm is one of endogenous pathological factors in TCM. Phlegm begins to appear in the body when the body fluid is not transported by vital energy normally and accumulates in certain parts of the body after being condensed.
Meridian energy	Meridians are channels that form a network in the body through which *qi* and blood (vital energy) flow [16, 17]. Meridian energy refers to the energy flow throughout the body via meridian vessels. The interconnected meridian vessels are composed of 12 meridians (lung, large intestine, stomach, heart, bladder, kidney, pericardium, small intestine, triple energizer, spleen, gallbladder, and liver) and collateral vessels. TCM views *qi* as essential for health maintenance and believes that *qi* should move uniformly and smoothly throughout the meridians. TCM practitioners improve health by modulating the balance of *qi* and blood in the body in order to achieve a balanced state of the human body, and thus disease can be prevented [28]. Many TCM studies have used a specific electrodermal device (Ryodoraku) to measure the meridian energy at 24 acupoints located on the 12 main

	meridians [29]. Body energy refers to the average energy flowing through the 12 meridians.
Health-related quality of life	Health-related quality of life (HRQOL) is an assessment of how the individual's well-being may be affected over time by a disease or disorder [30]. The SF-36 measured HRQOL by yielding two summary components composed of eight domains. Physical component scores comprise domains of physical functioning, role-physical, bodily pain, and general health. Mental component scores comprise domains of vitality, social functioning, role-emotional, and mental health [31].
