# Supplementary material for: The Effectiveness of a Traditional Chinese Medicine–Based Mobile Health App for Individuals With Prediabetes: Randomized Controlled Trial
Source: JMIR Mhealth Uhealth. 2023 Jun 20;11:e41099. doi: 10.2196/41099 (PMC10337399; doi:10.2196/41099)
Supplement: Multimedia Appendix 2 [file mhealth_v11i1e41099_app2.pdf]

Multimedia Appendix 2. Screenshot of the ordinary and TCM mHealth app

Modules	Screenshots		
Health diary	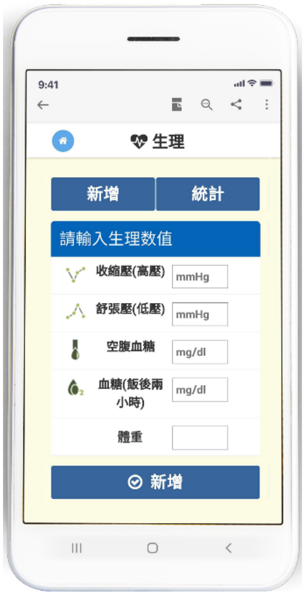	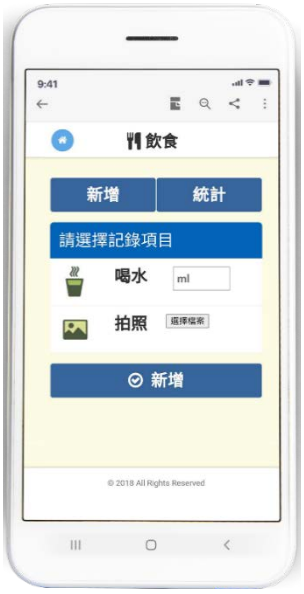	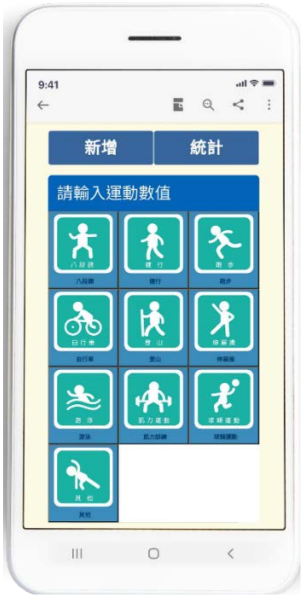
	The ordinary mHealth app		
Health education	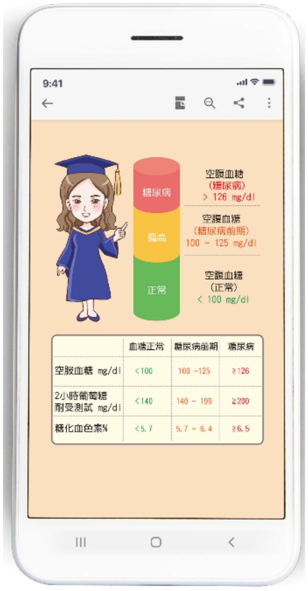	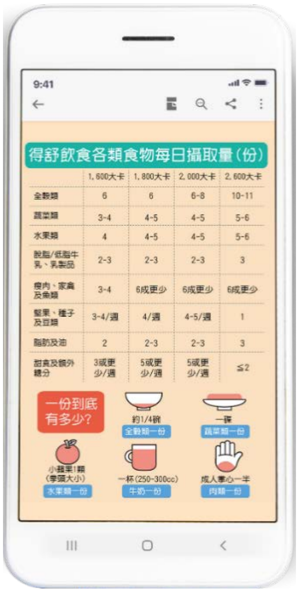	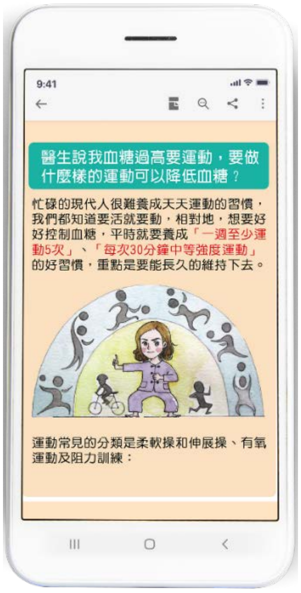
	The TCM mHealth app		

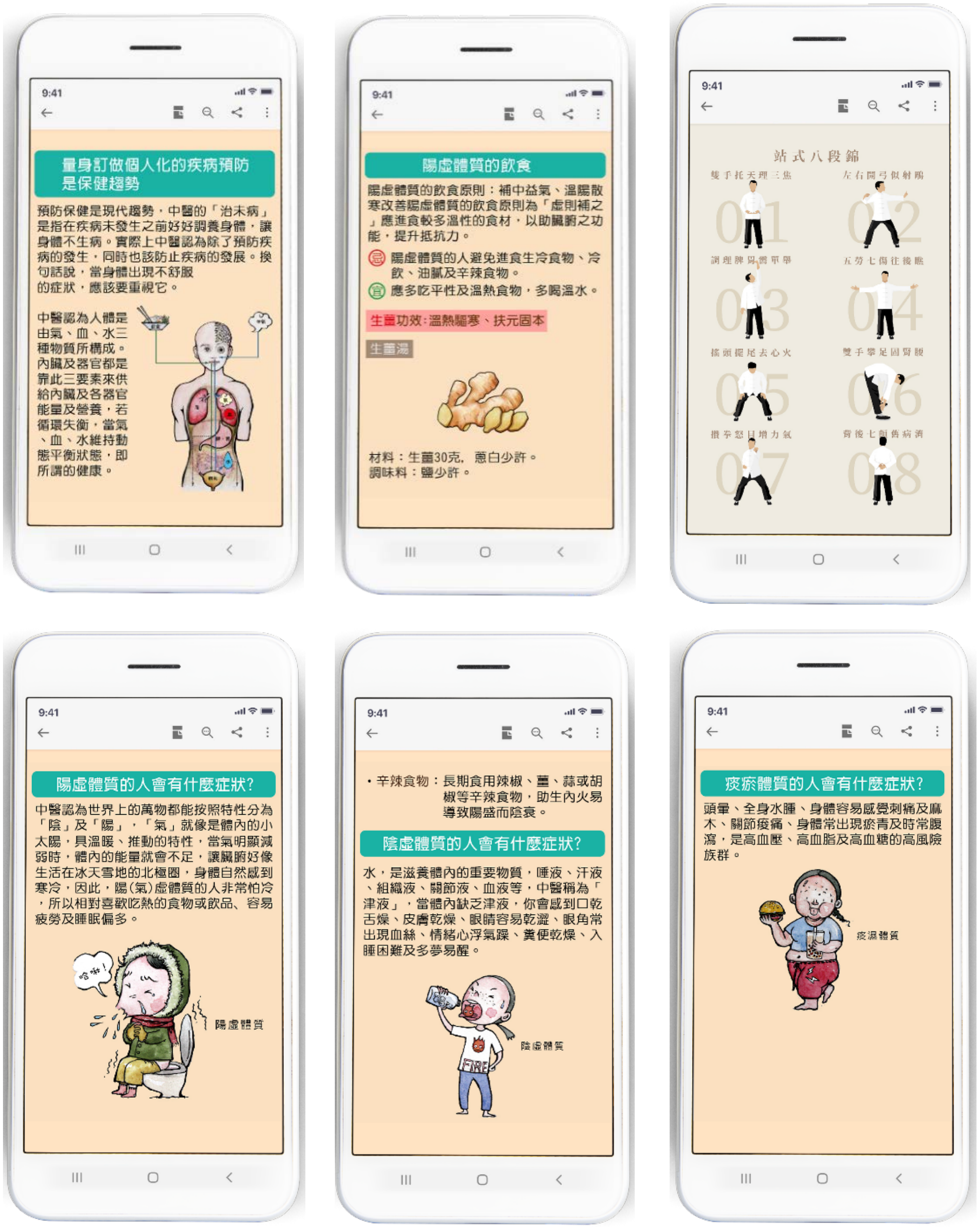
9:41
量身訂做個人化的疾病預防是保健趨勢
預防保健是現代趨勢，中醫的「治未病」是指在疾病未發生之前好好調養身體，讓身體不生病。實際上中醫認為除了預防疾病的發生，同時也該防止疾病的發展。換句話說，當身體出現不舒服的症狀，應該要重視它。
中醫認為人體是由氣、血、水三種物質所構成。內臟及器官都是靠此三要素來供給內臟及各器官能量及營養，若循環失衡，當氣、血、水維持動態平衡狀態，即所謂的健康。
9:41
陽虛體質的飲食
陽虛體質的飲食原則：補中益氣、溫陽散寒改善陽虛體質的飲食原則為「虛則補之」應進食較多溫性的食材，以助臟腑之功能，提升抵抗力。
忌 陽虛體質的人避免進食生冷食物、冷飲、油膩及辛辣食物。
宜 應多吃平性及溫熱食物，多喝溫水。
生薑功效：溫熱驅寒、扶元固本
生薑湯
材料：生薑30克，蔥白少許。
調味料：鹽少許。
9:41
站式八段錦
雙手托天理三焦
左右開弓似射鵰
五勞七傷往後瞧
搖頭擺尾去心火
雙手攀足固腎腰
攢拳怒目增力氣
01
02
03
04
05
06
07
08
9:41
陽虛體質的人會有什麼症狀?
中醫認為世界上的萬物都能按照特性分為「陰」及「陽」，「氣」就像是體內的小太陽，具溫暖、推動的特性，當氣明顯減弱時，體內的能量就會不足，讓臟腑好像生活在冰天雪地的北極圈，身體自然感到寒冷，因此，陽(氣)虛體質的人非常怕冷，所以相對喜歡吃熱的食物或飲品、容易疲勞及睡眠偏多。
哈啾！
陽虛體質
9:41
• 辛辣食物：長期食用辣椒、薑、蒜或胡椒等辛辣食物，助生內火易導致陽盛而陰衰。
陰虛體質的人會有什麼症狀?
水，是滋養體內的重要物質，唾液、汗液、組織液、關節液、血液等，中醫稱為「津液」，當體內缺乏津液，你會感到口乾舌燥、皮膚乾燥、眼睛容易乾澀、眼角常出現血絲、情緒心浮氣躁、糞便乾燥、入睡困難及多夢易醒。
FIRE
陰虛體質
9:41
痰瘀體質的人會有什麼症狀?
頭暈、全身水腫、身體容易感覺刺痛及麻木、關節痠痛、身體常出現瘀青及時常腹瀉，是高血壓、高血脂及高血糖的高風險族群。
痰濕體質

Milestone

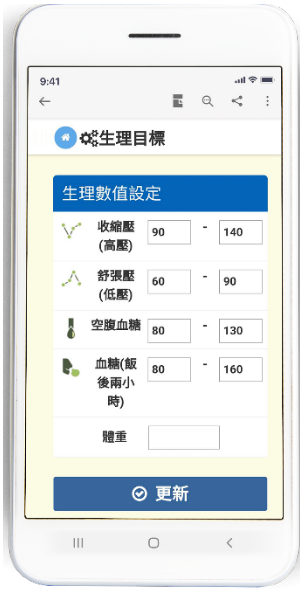

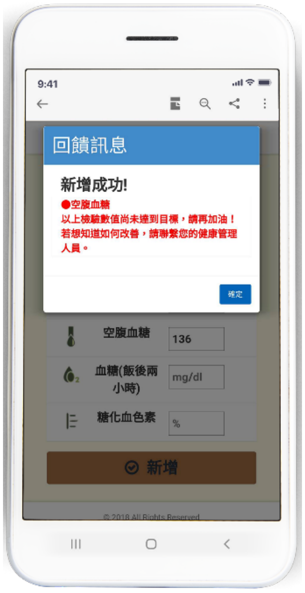

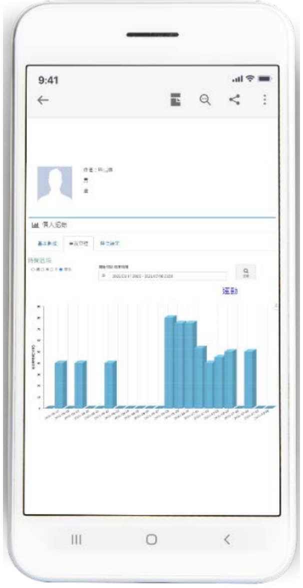

Chatroom

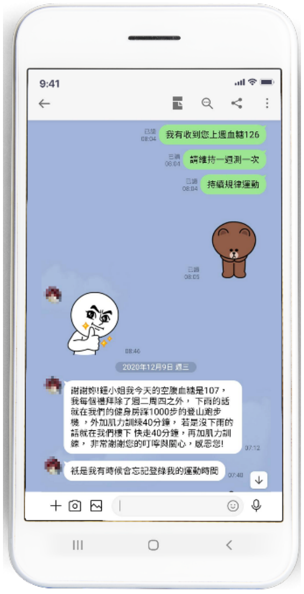

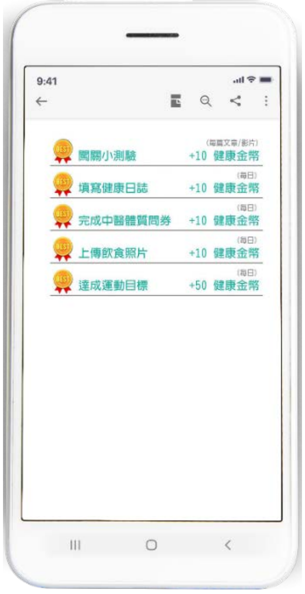

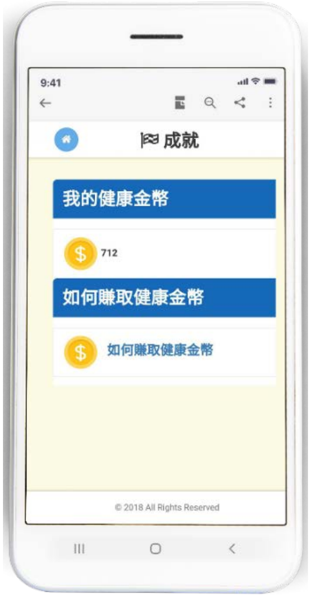

TCM: traditional Chinese medicine.
