# Supplementary material for: The Effectiveness of a Traditional Chinese Medicine–Based Mobile Health App for Individuals With Prediabetes: Randomized Controlled Trial
Source: JMIR Mhealth Uhealth. 2023 Jun 20;11:e41099. doi: 10.2196/41099 (PMC10337399; doi:10.2196/41099)
Supplement: Multimedia Appendix 3 [file mhealth_v11i1e41099_app3.pdf]

Multimedia Appendix 3. The mHealth app that maps behavior change technique taxonomy

Behavior Change Technique taxonomy	Intervention component and description
Goal setting (behavior)	Diet<br>· OMG: DASH diet (low-salt, low-fat dairy products, high-fiber fruits, and vegetables)<br>· TCMG: When following the DASH diet, appropriate foods should be chosen according to the participants' body constitution. The TCM mHealth app additionally suggests health education topics on body constitution-based diet advices. For example, participants with *yang*-deficiency body constitution were advised to eat warm and hot foods to supplement *qi*, such as ginger; those with *yin*-deficiency body constitution can eat cold and cool foods such as mushroom to avoid blood and interstitial fluid deficiency; participants with phlegm-stasis body constitution can eat kelp to promote *qi*-blood circulation<br>Physical activity<br>· OMG: Moderate intensity PA≥150 minutes/week such as brisk walking<br>· TCMG: The same goal as that of the OMG is followed. TCM PA advices used in the TCM mHealth app is mainly kind of PA to avoid and to practice based on their body constitution. Qigong including belly breathing and Baduanjin were recommended for all participants since they can improve *qi* and are appropriate for all types of body constitutions. Individuals with *yin*-deficiency body constitution were advised to avoid vigorous PA.
Goal setting (outcome)	Set blood sugar level or weight goal as an outcome of changed PA and dietary behavior
Discrepancy between current behavior and goal	Assess the charts over a week or month to compare the discrepancy between the actual and desired behavior at different time points
Review outcome goal (s)	Examine changes in weight and blood sugar levels and consider adjusting outcome goal (s)
Feedback on behavior	Participants upload data (e.g., PA), check the charts over a week or month in the mHealth app and receive text messages from the researchers on the number of minutes spent on PA per week
Self-monitoring of behavior	Track the participant's PA and dietary behavior
Self-monitoring of outcome of behavior	Track the participants' weight, BMI, and blood sugar levels
Feedback on outcome (s) of behavior	Participants upload data (e.g., FPG); when the data exceed the setting range, a window pops up as a reminder. They can check the charts over a week or month in the mHealth app
Social support (unspecified)	The researchers sent text messages to the participants in a personal chat room, provided feedback on the results, and encouraged participants to share their experiences in the group chat room.
Information about health consequences	· OMG: Demonstrate DASH diet and regular exercise control blood sugar and prevent prediabetes<br>· TCMG: Explain that a body constitution-based diet and PA advices can stimulate the *qi*-blood circulation to control blood sugar

	levels
Social comparison	Announce weekly leaderboard reports of participants' PA in the group chat room and make it available to others for comparison
Prompts/cue	The researchers monitored logins and log file analysis at least once every week in the mHealth app backend. If the participants did not use the app, complete the diary, and watch health education, additional text messages were sent to the participants. A pop-up window will remind participants in the mHealth app if the data (e.g., blood sugar level) exceeds the setting range
Credible source	Our goals refer to moderate intensity PA ≥ 150 minutes/week recommended by the ADA and the recommended DASH diet from the USDA
Material reward	Participants receive virtual gold from the mHealth app and claim actual prizes from the researchers
Non-specific reward	Participants can pass the challenge with a point-based reward in the mHealth app to gain a virtual gold
Social reward	The researcher sends participants a text message when achieving the goal. For example, "Good job, the goal for this week has been achieved; please keep going."
Reward (outcome)	Gain virtual gold if the participants achieve the weight loss or control blood sugar level goals
Focus on past success	The mHealth provided the charts over a week or month, and the participants could compare past to present behavior (outcome)

ADA, American Diabetes Association; BMI, body mass index; DASH, Dietary Approaches to Stop Hypertension; FPG, fasting plasma glucose; OMG, ordinary mHealth app group; PA, physical activity; TCM, traditional Chinese medicine; TCMG, traditional Chinese medicine mHealth app group; USDA, United States Department of Agriculture.
