# Supplementary material for: The Effectiveness of a Traditional Chinese Medicine–Based Mobile Health App for Individuals With Prediabetes: Randomized Controlled Trial
Source: JMIR Mhealth Uhealth. 2023 Jun 20;11:e41099. doi: 10.2196/41099 (PMC10337399; doi:10.2196/41099)
Supplement: Multimedia Appendix 4 [file mhealth_v11i1e41099_app4.pdf]

CONSORT-EHEALTH (V 1.6.1) -Submission/Publication Form

Background

* Your name
Hsueh-Wen Chung

* Primary Affiliation (short), City, Country
University of Yang Ming Chiao Tung, Taipei, Taiwan

* Your e-mail address
snow721103@gmail.com

* Title of your manuscript
The Effectiveness of a Traditional Chinese Medicine-based Mobile Health Application for Individuals with Prediabetes: A Randomized Controlled Trial

* Name of your App/Software/Intervention
TCM App (traditional Chinese medicine application)

* Language(s)
Traditional Chinese

* Accessibility
Access only for special user groups, not open

* Primary Medical Indication/Disease/Condition
Prediabetes

* Primary Outcomes measured in trial
Blood sugar control, body constitution, meridian energy, and health-related quality of life

* Secondary/other outcomes
Body mass index, dietary behavior, and physical activity

* Recommended "Dose"
Approximately Daily

* Approx. Percentage of Users (starters) still using the app as recommended after 3 months
31-40%

* Overall, was the app/intervention effective?
Partly: SOME primary outcomes were significantly better in intervention group vs control

* Article Preparation Status/Stage

Submitted to a journal and after receiving initial reviewer comments

* Journal
JMIR mHealth and uHealth

* Is this a full powered effectiveness trial or a pilot/feasibility trial?
Fully powered

* Manuscript tracking number *
41099

TITLE AND ABSTRACT

1a) TITLE: Identification as a randomized trial in the title

Does your paper address CONSORT item 1a? Yes

1a-i) Identify the mode of delivery in the title 5

✓ Does your paper address subitem 1a-i?

Yes. We have described it in the title of the manuscript: The Effectiveness of a Traditional Chinese Medicine-based "Mobile Health Application" for Individuals with Prediabetes: A Randomized Controlled Trial (page 1)

1a-ii) Non-web-based components or important co-interventions in title 1

✓ Does your paper address subitem 1a-ii?

Not applicable. We omitted a co-intervention in this study.

1a-iii) Primary condition or target group in the title 5

✓ Does your paper address subitem 1a-iii?

Yes. We have described it in the title of the manuscript: The Effectiveness of a Traditional Chinese Medicine (TCM)-based Mobile Health Application for "Individuals with Prediabetes": A Randomized Controlled Trial (page 1)

1b) ABSTRACT: Structured summary of trial design, methods, results, and conclusions

1b-i) Key features/functionalities/components of the intervention and comparator in the METHODS section of the ABSTRACT 5

✓ Does your paper address subitem 1b-i?

Yes. We have described it in the Methods section of the Abstract: "The ordinary mHealth app included physical activity (PA), diet, disease education, and individual records. The TCM mHealth app additionally included *qi* and body constitution as well as constitution-based PA and diet advices. The CG received the usual care alone and did not use apps." (Methods/Abstract, page 2)

1b-ii) Level of human involvement in the METHODS section of the ABSTRACT 1

✓ Does your paper address subitem 1b-ii?

No. We did not mention it in the Methods section of the Abstract.

1b-iii) Open vs. closed, web-based (self-assessment) vs. face-to-face assessments in the METHODS section of the ABSTRACT 2

✓ Does your paper address subitem 1b-iii?

No. We did not mention it in the Methods section of the Abstract.

1b-iv) RESULTS section in abstract must contain use data 4

✓ Does your paper address subitem 1b-iv?
Yes. We have described it in the Methods section of the Abstract: "The participants were randomly assigned to TCM mHealth app (TCMG; n=42), ordinary mHealth app (OMG; n=41), or control (CG; n=38) group." (Methods/Abstract, page 2)

1b-v) CONCLUSIONS/DISCUSSION in abstract for negative trials 4

✓ 1b-v) CONCLUSIONS/DISCUSSION in abstract for negative trials
Yes. We described it in the Results section of the Abstract: "There were no significant differences in fasting plasma glucose, *yin*-deficiency body constitution, Dietary Approaches to Stop Hypertension dietary behavior, and total PA among the three groups after the intervention." (Conclusion/Abstract, page 3)

Introduction

2a) In INTRODUCTION: Scientific background and explanation of rationale

2a-i) Problem and the type of system/solution 5

✓ Problem and the type of system/solution
Yes. We have described it in the Introduction: "To the best of our knowledge, no study has been conducted to identify whether TCM-based health concepts could be incorporated into mobile health application (mHealth app) for individuals with prediabetes." (sixth paragraphic, Introduction, page 6)

2a-ii) Scientific background, rationale: What is known about the (type of) system 5

✓ Does your paper address subitem 2a-ii?
Yes. We have described it in the Introduction section (second, third, fourth, and fifth paragraph, Introduction, pages 4–6)

2b) In INTRODUCTION: Specific objectives or hypotheses

✓ Does your paper address CONSORT subitem 2b?
Yes. We have described it in the Introduction: "This study aimed to develop a TCM mHealth app and examine its effectiveness on blood sugar control, body constitution, body energy, and health-related quality of life [HRQOL] (primary outcomes); and BMI, dietary behavior, and PA (secondary outcomes) among individuals with prediabetes. The study assumption was that the TCM mHealth app would improve overall health through health behavior and BMI. Therefore, primary outcomes were overall health indicators (including body constitution and meridian energy), and secondary outcomes were BMI and health behaviors." (sixth paragraph, Introduction, page 6-7)

Methods

3a) Description of trial design (such as parallel, factorial) including allocation Ratio

✓ Does your paper address CONSORT subitem 3a?
Yes. We have described it in the Study design subsection of the Methods section in the main manuscript: "This study was an open-label, parallel-group RCT (ClinicalTrials.gov NCT04096989) with a three-group design." (Study design/Methods, page 7)

3b) Important changes to methods after trial commencement (such as eligibility criteria), with reasons

✓ Important changes to methods after trial commencement (such as

eligibility criteria), with reasons
No important changes to the Methods were made after the trial commencement.

3b-i) Bug fixes, Downtimes, Content Changes 5

✓ Bug fixes, Downtimes, Content Changes

No bug fixes, downtimes, or content-related changes were needed in this trial.

4a) Eligibility criteria for participants

✓ Eligibility criteria for participants

Yes. We have described it in the Study design subsection of the Methods section: “The inclusion criteria were (a) having been diagnosed with prediabetes (with an HbA1c of 5.7% to 6.4% or an FPG level of 100 mg/dL to 125 mg/dL [2]); (b) aged 20 years and above; (c) not having cardiopulmonary disease, cancer, or other major diseases, and (d) who had provided informed consent. Those who had used hypoglycemic agents, β-blockers, thiazide diuretics, nicotinic acid, or steroids within the past 3 months were excluded.” (Study design/Methods, page 7)

4a-i) Computer / Internet literacy 3

✓ Does your paper address subitem 4a-i?

No. We did not clarify the computer/internet literacy in this main text because the participants were all individuals who used their own smartphones daily.

4a-ii) Open vs. closed, web-based vs. face-to-face assessments: 3

✓ Does your paper address subitem 4a-ii?

We did not describe it in the main text. We have added an explanation here: “We recruited the participants by displaying advertisements through a poster at our hospital as well as local clinics, and by announcing on social media such as Facebook and LINE. Initial eligibility was confirmed by laboratory testing using the electronic medical record. If eligible, the researcher offered a brief outline of the study face-to-face or over a telephone call.”

4a-iii) Information giving during recruitment 3

No. It is not described in the main text.

4b) Settings and locations where the data were collected

✓ Does your paper address CONSORT subitem 4b?

Yes. We have described those details in the Study design and Data collection subsections of the Methods section: “We cooperated with the health examination center and outpatient clinics at a teaching hospital in northern Taiwan to recruit individuals with prediabetes…” (Study design and Data collection/Methods, page 7)

4b-i) Report if outcomes were (self-)assessed through online questionnaires 4

✓ Does your paper address subitem 4b-i?

We did not describe it in the main text. We have added an explanation here: “All participants were required to follow instructions by completing a baseline questionnaire. The two follow-up evaluations were completed via a web-based instrument or paper-based questionnaire.”

4b-ii) Report how institutional affiliations are displayed 1

✓ Does your paper address subitem 4b-ii?
No. It is not described in the main text.

5) The interventions for each group with sufficient details to allow replication, including how and when they were actually administered

5-i) Mention names, credential, affiliations of the developers, sponsors, and Owners 4

✓ Does your paper address subitem 5-i?
We did not describe it in the main text. We have added an explanation here: "The intervention development process involved a collaboration from app developers (National Yang Ming Chiao Tung University Institute of BioMedical Informations)."

5-ii) Describe the history/development process 5

✓ Does your paper address subitem 5-ii?
Yes. We have described it in the Intervention subsection of the Methods section (first paragraph, Intervention/Method, pages 9).

5-iii) Revisions and updating 5

✓ Does your paper address subitem 5-iii?
We did not update the mHealth app during the intervention.

5-iv) Quality assurance methods 3

✓ Does your paper address subitem 5-iv?
Yes. We have described it in the Intervention subsection of the Methods section: "The researchers monitored logins and log file analysis at least once every week at the mHealth app back-end. If the participants did not use the app, complete the diary, or watch health education, additional text messages were sent to the participants." (third paragraph, Intervention/Method, page 11)

5-v) Ensure replicability by publishing the source code, and/or providing screenshots/screen-capture video, and/or providing flowcharts of the algorithms used 3

✓ Does your paper address subitem 5-v?
Yes. We have provided the screenshots in Multimedia Appendix 2.

5-vi) Digital preservation 1

✓ Does your paper address subitem 5-vi?
No. We did not include the website of the mHealth app program in the main text.

5-vii) Access 3

✓ Does your paper address subitem 5-vii?
We did not describe it in the main text. We have added an explanation here: "The mHealth app was completely online and developed for smartphone use. Participants, therefore, required access to the internet and a smartphone to use the mHealth app."

5-viii) Mode of delivery, features/functionalities/components of the intervention and comparator, and the theoretical framework 5

✓ Does your paper address subitem 5-viii?
Yes. We have described it in the Intervention subsection of the Methods section (second and third paragraphs, Intervention/Method, pages 9-11).

5-ix) Describe use parameters 3

✓ Does your paper address subitem 5-ix?

We did not describe it in the Methods section, but we mentioned it in the Limitations section: "…the use of the mHealth apps in the follow-up period was not monitored. In addition, during the intervention period, the participants were reminded to use the apps but the time spent on app use varied, suggesting that the participation level may be different." (Limitations, page 24)

5-x) Clarify the level of human involvement 4

✓Does your paper address subitem 5-x?

Yes. We have described it in the Intervention subsection of the Methods section: "The researchers monitored logins and log file analysis at least once every week at the mHealth app back-end. If the participants did not use the app, complete the diary, or watch health education, additional text messages were sent to the participants." (third paragraph, Intervention/Methods, page 10)

5-xi) Report any prompts/reminders used

✓Does your paper address subitem 5-xi?

Yes. We have described it in the Intervention subsection of the Methods section: "A text message was sent to the participants to remind them of reading the topics every week. (page 8) and "…the participants set a goal of an FPG level of 60 to 99 mg/dL. When this goal was achieved, a window would pop up to show the achievement." (second paragraph, Intervention/Methods, page 9-10)

5-xii) Describe any co-interventions (incl. training/support")

✓5-xii) Describe any co-interventions (incl. training/support)

Not applicable. No co-interventions were used.

6a) Completely defined pre-specified primary and secondary outcome measures, including how and when they were assessed

✓Does your paper address CONSORT subitem 6a?

Yes. We have described it in the Data collection subsection of the Methods section: "Primary outcome measures, including blood sugar control (FPG and HbA1c levels), body constitution, meridian energy, and HRQOL, were collected at baseline (T1), the end of intervention (T2), and 1 month after the intervention (T3)." (page 11) and "The secondary outcomes in this study were BMI, dietary behavior, and PA." (Data collection/Methods, page 12)

6a-i) Online questionnaires: describe if they were validated for online use and apply CHERRIES items to describe how the questionnaires were designed/deployed

✓Does your paper address subitem 6a-i?

No. We did not apply CHERRIES items to describe the designed questionnaires. However, the trial was reported in accordance with the CONSORT-EHEALTH checklist.

6a-ii) Describe whether and how "use" (including intensity of use/dosage) was defined/measured/monitored 5

✓Does your paper address subitem 6a-ii?

Yes. We have described it in the Intervention subsection of the Methods section: "The researchers monitored logins and log file analysis at least once every week at the mHealth app back-end. If the participants did not

use the app, complete the diary, or watch health education, additional text messages were sent to the participants." (the third paraph, Intervention/Methods, page 11)

6a-iii) Describe whether, how, and when qualitative feedback from participants was obtained 3

✓Does your paper address subitem 6a-iii?

Yes. We have described it in the Intervention subsection of the Methods section: "The researchers sent text messages to the participants in the personal chat room, provided feedback on the results, and encouraged participants to share their experiences in the group chat room." (second paragraph, Intervention/Methods, pages 10)

6b) Any changes to trial outcomes after the trial commenced, with reasons

✓ Does your paper address CONSORT subitem 6b?

There were no changes to trial outcomes after the trial commenced.

7a) How sample size was determined

7a-i) Describe whether and how expected attrition was taken into account when calculating the sample size 5

✓ Does your paper address subitem 7a-i?

Yes. We have described it in the Participants subsection of the Methods section: "The required sample size was calculated on the basis of repeated-measures analysis of variance (ANOVA), α=0.05, power=0.80, effect size= 0.3 [32], and three repeated measurements as per a previous study, use of G-power indicated that the required sample size was 31 per group. To account for 30% attrition [34], we recruited 121 participants in the study. " (first paragraph, Participants/Methods, page 8)

7b) When applicable, explanation of any interim analyses and stopping Guidelines

✓ Does your paper address CONSORT subitem 7b?

We did not perform any interim analyses and stopping guidelines in this study.

8a) Method used to generate the random allocation sequence

Yes. We have described it in the Study design subsection of the Methods section: "A statistician drew up a computer-generated randomization list." (second paragraph, Study design/Methods, page 7)

8b) Type of randomization; details of any restriction (such as blocking and block size)

✓ Does your paper address CONSORT subitem 8b?

We did not describe the type of randomization because "the trial involved a simple randomization." (page 7)

9) Mechanism used to implement the random allocation sequence (such as sequentially numbered containers), describing any steps taken to conceal the sequence until interventions were assigned

✓ Does your paper address CONSORT subitem 9?

Yes. We have described it in the Study design subsection of the Methods section: "The allocation sequence was kept in an opaque, sealed, and stapled envelope, and a staff member in the outpatient clinic who was not involved

in the study, held the sealed envelopes. After the participants agreed to participate in the study, the researcher opened the envelope to reveal their group assignment." (second paragraph, Study design/Methods, page 7)

10) Who generated the random allocation sequence, who enrolled participants, and who assigned participants to interventions

✓ Does your paper address CONSORT subitem 10?

Yes. We have described it in the Study design subsection of the Methods section: "A statistician drew up a computer-generated randomization list. The allocation sequence was kept in an opaque, sealed, and stapled envelope, and a staff member in the outpatient clinic who was not involved in the study, held the sealed envelopes. After the participants agreed to participate in the study, the researcher opened the envelope to reveal their group assignment." (second paragraph, Study design/Methods, page 7–8)

11a) If done, who was blinded after assignment to interventions (for example, participants, care providers, those assessing outcomes) and how

11a-i) Specify who was blinded, and who wasn't 5

No. We did not mention it in the Methods section because the study was an open-label trial.

11a-ii) Discuss e.g., whether participants knew which intervention was the "intervention of interest" and which one was the "comparator"

✓ Does your paper address subitem 11a-i?

Not applicable. There was no similarity of interventions in this study.

11b) If relevant, description of the similarity of interventions

✓ Does your paper address CONSORT subitem 11b?

Not applicable. There was no similarity of interventions in this study.

12a) Statistical methods used to compare groups for primary and secondary Outcomes

✓ Does your paper address CONSORT subitem 12a?

Yes. We have described it in the Statistical analysis subsection of the Methods section: "We used the paired t-test to examine the changes in outcome variables within groups. One-way ANOVA was used for comparisons among groups with the Scheffe's post-hoc test being used for pairwise comparisons. Finally, we used the generalized estimating equations (GEE) to estimate the intervention effects after adjusting for age, gender, and baseline value of the outcome variable." (second paragraph, Statistical analysis/Methods, page 13)

12a-i) Imputation techniques to deal with attrition / missing values 5

✓ Does your paper address subitem 12a-i?

Yes. We have described it in the Statistical analysis subsection of the Methods section: "We analyzed the data using an intention-to-treat analysis. For participants with incomplete or missing data, we used the maximum likelihood method for imputation [48]." (first paragraph, Statistical analysis/Methods, pages 13)

12b) Methods for additional analyses, such as subgroup analyses and adjusted analyses

✓ Does your paper address CONSORT subitem 12b?

Yes. We have described it in the Statistical Analysis subsection of the

Methods section: "One-way ANOVA was used for comparisons among groups with the Scheffe's post-hoc test being used for pairwise comparisons."(page 12) and "...we used the generalized estimating equations (GEE) to estimate the intervention effects after adjusting for age, gender, and baseline value of the outcome variable." (page 13)

X26) REB/IRB Approval and Ethical Considerations [recommended as subheading under "Methods"] (not a CONSORT item)

X26-i) Comment on ethics committee approval

✓ Does your paper address subitem X26-i? 5

Yes. We have described it in the Study design subsection of the Methods section: "This study was approved by an institutional review board at the Taipei Tzu Chi Hospital (Approval No.: 08-X-026)." (third paragraph, Study design/Methods, page 8)

x26-ii) Outline informed consent procedures 5

✓ Does your paper address subitem X26-ii?

Yes. We have described it in the Study design subsection of the Methods section: "Informed consent form was signed by all participants before enrollment in the study." (third paragraph, Study design/Methods, page 8)

X26-iii) Safety and security procedures 3

✓ Does your paper address subitem X26-iii?

Yes. We have described it in the Study design subsection of the Methods section: "The informed consent form stated that the risk of participation in this study was low. If the participants felt physically or mentally unwell due to their participation, they had to contact the researchers and had the right to withdraw at any time. No adverse events were reported during the study period." (third paragraph, Study design/Methods, page 8)

Results

13a) For each group, the numbers of participants who were randomly assigned, received intended treatment, and were analyzed for the primary outcome

✓ Does your paper address CONSORT subitem 13a?

Yes. We have described it in Figure 1 and in the Participant subsection of the Methods section (second paragraph, Participant/Methods, page 13).

13b) For each group, losses and exclusions after randomization, together with Reasons

✓ Does your paper address CONSORT subitem 13b? (NOTE: Preferably, this is shown in a CONSORT flow diagram)

Yes. We have described it in Figure 1.

13b-i) Attrition diagram 5

✓ Does your paper address subitem 13b-i?

Yes. We have mentioned it in Figure 1.

14a) Dates defining the periods of recruitment and follow-up

✓Does your paper address CONSORT subitem 14a?

Yes. We have described it in the Study design and Data collection subsection of the Methods section: "...to recruit individuals with prediabetes from February 2020 to May 2021." (page 7) and "...were

collected at baseline (T1), the end of intervention (T2), and 1 month after the intervention (T3)." (Study design and Data collection/Methods, page 11)

14a-i) Indicate if critical "secular events" fell into the study period 1

✓ Does your paper address subitem 14a-i?

Not applicable. We did not mention it in the main text.

14b) Why the trial ended or was stopped (early)

✓Does your paper address CONSORT subitem 14b?

Not applicable. There was no interruption or discontinuation during the intervention.

15) A table showing baseline demographic and clinical characteristics for each group

✓Does your paper address CONSORT subitem 15?

Yes. We have described it in Table 1 and in the Characteristics of the study participants subsection of the Results section (first paragraphic, Characteristics of the study participants/Results, page 13)

15-i) Repot demographics associated with digital divide issues 3

✓ Does your paper address subitem 15-i?

No. We did not mention it in the main text.

16) For each group, number of participants (denominator) included in each analysis and whether the analysis was by original assigned groups

16-i) Repot multiple "denominators" and provide definitions 5

✓ Report multiple "denominators" and provide definitions

Yes. We have described all the denominators in the manuscript, figures, and tables.

16-ii) Primary analysis should be intent-to-treat 5

✓Does your paper address subitem 16-ii?

Yes. We used ITT to analyze the data in this study.

17a) For each primary and secondary outcome, results for each group, and the estimated effect size and its precision (such as 95% confidence interval)

✓Does your paper address CONSORT subitem 17a?

Yes. In the Results section, we have described outcomes with mean, standard deviation, p-value, and 95% confidence interval.

17a-i) Presentation of process outcomes such as metrics of use and intensity of use 5

✓17a-i) Presentation of process outcomes such as metrics of use and intensity of use

Yes. We have described them in the Primary and secondary outcomes of the Results (pages 14–21)

17b) For binary outcomes, presentation of both absolute and relative effect sizes is recommended

✓Does your paper address CONSORT subitem 17b?

No. We did not mention it because we did not use binary outcomes in the study.

18) Results of any other analyses performed, including subgroup analyses and

adjusted analyses, distinguishing pre-specified from exploratory

✓ Does your paper address CONSORT subitem 18?
Yes. We have described the GEE model in the Primary and secondary outcomes of the Results section (pages 14–21)

18-i) Subgroup analysis of comparing only users 1

✓ Does your paper address subitem 18-i?
Not applicable. We did not mention it in the main text.

19) All important harms or unintended effects in each group

✓ Does your paper address CONSORT subitem 19?
We did not have any/unintended effects.

19-i) Include privacy breaches, technical problems 1

✓ Does your paper address subitem 19-i?
We did not experience privacy breaches or technical challenges during the intervention.

19-ii) Include qualitative feedback from participants or observations from staff/researchers

✓ Does your paper address subitem 19-ii?
We did not have specific problems during the intervention.

Discussion

22) Interpretation consistent with results, balancing benefits and harms, and considering other relevant evidence

22-i) Restate study questions and summarize the answers suggested by the data, starting with primary outcomes and process outcomes (use)5

✓Does your paper address subitem 22-i?
Yes. We have described it in the Discussion (first paragraphic, Discussion, pages 21)

22-ii) Highlight unanswered new questions, suggest future research. 5

✓Does your paper address subitem 22-ii?
Yes. We have described it in the Discussion section: "Further studies are needed to examine the inter-relationships among those outcomes." (first pagraphic, Discussion, page 21), "The universal improvement in health behavior may be the reason for the lack of significant effect on those outcomes when the ordinary mHealth app group was compared to the controls." (second paragraph, Discussion, page 22), "The results imply that using the TCM mHealth app can better improve body energy and HRQOL in individuals with prediabetes compared to the ordinary mHealth app. This may be because people who practice TCM consider that health conditions can improve with sufficient *qi*. The importance of these indicators across cultures needs to be examined in future studies." (fourth paragraph, Discussion, page 22-23), "In future studies, we suggest that more than two tests should be used to enhance diagnostic accuracy." (sixth paragraph, Discussion, page 23), and "More study is needed to develop effective interventions to improve the *yin*-deficiency body constitution among individuals with prediabetes." (seventh paragraph, Discussion, page 23)

20) Trial limitations, addressing sources of potential bias, imprecision, and, if relevant, multiplicity of analyses

20-i) Typical limitations in ehealth trials

✓ Does your paper address subitem 20-i?

Yes. We have described it in the Limitations section (first paragraph, Limitation, page 24)

21) Generalisability (external validity, applicability) of the trial findings

21-i) Generalizability to other populations

✓Does your paper address subitem 21-i?5

Yes. We have described it in the Conclusion section of the manuscript (Conclusion, page 25)

21-ii) Discuss if there were elements in the RCT that would be different in a routine application setting

✓Does your paper address subitem 21-ii?

No. We did not mention it in the main text.

OTHER INFORMATION

23) Registration number and name of trial registry

✓ Does your paper address CONSORT subitem 23?

Yes. We have mentioned it in the study design section of the methods section: “Trial Registration: ClinicalTrials.gov NCT04096989” (page 3)

24) Where the full trial protocol can be accessed, if available

✓ Does your paper address CONSORT subitem 24?

Yes. We have mentioned it in the abstract: “https://clinicaltrials.gov/ct2/show/NCT04096989”

25) Sources of funding and other support (such as supply of drugs), role of funders

✓ Does your paper address CONSORT subitem 25?

Yes. We have provided a statement in the acknowledgments section: “This study was funded by the Taipei Tzu Chi Hospital, Buddhist Tzu Chi Medical Foundation (TCRD-TPE-109-05). The publication fee for this manuscript is funded by the National Science and Technology Council, Taiwan (MOST 111-2314-B-A49-011-MY3)” (page 25)

X27) Conflicts of Interest (not a CONSORT item)

X27-i) State the relation of the study team towards the system being evaluated 5

✓ Does your paper address subitem X27-i?

The authors declare no conflict of interest. (page 25)
