# Supplementary material for: The Effectiveness of a Traditional Chinese Medicine–Based Mobile Health App for Individuals With Prediabetes: Randomized Controlled Trial
Source: JMIR Mhealth Uhealth. 2023 Jun 20;11:e41099. doi: 10.2196/41099 (PMC10337399; doi:10.2196/41099)
Supplement: Multimedia Appendix 5 [file mhealth_v11i1e41099_app5.pdf]

Multimedia Appendix 5. Comparison of the primary and secondary outcomes among the TCM mHealth app, ordinary mHealth app, and control groups (n=121)

Variables		Group	Pre-intervention (T1) Mean±SD	Post-intervention (T2) Mean±SD	1 month after intervention (T3) Mean±SD	P^2	P^3
**Primary outcomes**							
Blood sugar control	Fasting plasma glucose	P^1 / Posthoc	.78	.52	.08		
		C	106.13±8.44	105.93±10.35	105.29±10.66	.91	.65
		O	106.29±8.51	104.91±9.41	108.62±12.54	.38	.25
		T	107.33±8.17	104.61±7.91	106.12±9.85	.12	.55
	HbA1c	P^1 / Posthoc	.36	.54	.59		
		C	6.09±0.25	6.03±0.25	6.02±0.31	.08	.04
		O	6.06±0.26	5.94±0.26	5.94±0.26	.001	<.001
		T	6.15±0.32	6.00±0.26	5.96±0.05	<.001	<.001
Body constitution	*Yang*-deficiency	P^1 / Posthoc	.02/ T>C	.84	.44		
		C	27.03±5.61	26.55±5.61	25.81±5.54	.60	.14
		O	27.71±6.21	26.41±5.82	26.03±5.25	.09	.06
		T	30.67±6.44	27.04±7.26	27.08±6.35	.003	.002
	Yin-deficiency	P^1 / Posthoc	.06	.92	.54		
		C	25.42±5.30	25.40±5.11	24.35±4.00	.98	.14
		O	25.90±5.67	25.86±5.76	24. 86±5.44	.96	.19
		T	28.36±6.80	26.04±7.00	25.66±6.80	.06	.02
	Phlegm-stasis	P^1 / Posthoc	.03/ T>C	.93	.67		
		C	21.53±5.17	22.37±5.05	21.92±5.98	.42	.69
		O	22.93±6.40	22.41±5.95	21.45±4.22	.60	.10
		T	25.14±6.09	22.53±6.48	22.24±5.25	.03	.004
Meridian energy	Body energy	P^1 / Posthoc	.63	.53	.13		
		C	41.40±21.87	40.25±18.48	42.52±23.59	.78	.80
		O	43.81±21.63	42.51±23.55	39.10±20.48	.75	.23
		T	39.34±19.63	46.79±17.89	48.27±21.97	.04	.03
Health-related quality of life	Physical component score	P^1 / Posthoc	.28	.45	.35		
		C	50.06±6.96	49.01±9.86	49.99±8.93	.35	.95
		O	49.37±6.34	50.88±6.08	52.20±4.35	.18	.01
		T	47.51±8.55	51.39±8.04	52.33±6.67	<.001	<.001
	Mental component score	P^1 / Posthoc	.07	.03/ T>C, O>C	.10		
		C	50.02±7.52	47.93±9.67	49.69±9.73	.16	.83
		O	49.41±8.90	51.94±7.55	51.76±7.42	.04	.05
		T	45.99±8.81	52.00±8.04	52.92±7.19	<.001	<.001
**Secondary outcomes**							
BMI		P^1 / Posthoc	.51	.52	.63		
		C	25.25±3.20	25.03±3.08	25.10±3.08	.07	.29
		O	26.36±4.93	26.12±5.06	25.97±5.06	.04	.001

	T	25.77±4.47	25.44±4.34	25.25±4.21	.003	<.001
DASH dietary behavior	P^1 / Posthoc	.72	.24	.06		
	C	36.66±5.05	38.23±5.54	38.73±5.05	.02	.01
	O	36.02±4.93	37.82±4.86	37.59±5.12	.001	.01
	T	36.98±6.16	39.77±4.67	40.04±4.41	<.001	<.001
Total physical activity	P^1 / Posthoc	.02/ O> T	.02/ O>C	.02/ O>C		
	C	687.07± 26.24	868.57± 1005.93	1086.59± 1202.56	.17	.04
	O	1344.50± 1798.14	1762.34± 2233.34	1957.21± 2273.54	.04	.01
	T	625.51± 1032.78	1055.61± 1236.45	1147.78± 1343.24	.02	.03

P^1, one-way ANOVA was used to examine differences among groups; P^2, paired t-test, was used to examine the change between pre-intervention and post-intervention; P^3, paired t-test, was used to examine the change between pre-intervention and 1 month after the intervention.
C, control group; O, ordinary mHealth app group; T, TCM mHealth app group.
SD, standard deviation.
