# Supplementary material for: The Effectiveness of a Traditional Chinese Medicine–Based Mobile Health App for Individuals With Prediabetes: Randomized Controlled Trial
Source: JMIR Mhealth Uhealth. 2023 Jun 20;11:e41099. doi: 10.2196/41099 (PMC10337399; doi:10.2196/41099)
Supplement: Multimedia Appendix 6 [file mhealth_v11i1e41099_app6.pdf]

Multimedia Appendix 6. Generalized estimating equation models to compare the differences among the three groups, using the control group as the reference

	β	SE	95%CI		*P*-value
			Lower	Upper	
**Fasting plasma glucose**					
Intercept	100.86	4.96	91.14	110.59	<.001
Age	0.08	0.08	−0.08	0.23	.33
Male (vs. female)	1.47	1.36	−1.20	4.15	.28
Group					
OMG (vs. CG)	0.42	1.88	−3.26	4.11	.82
TCMG (vs. CG)	1.63	1.88	−2.01	5.26	.38
Time					
T2 (vs. T1)	−0.20	1.81	−3.76	3.35	.91
T3 (vs. T1)	−0.84	1.84	−4.48	2.79	.65
*Group*Time*					
OMG * T2 (vs. CG * T1)	−1.18	2.42	−5.92	3.56	.62
OMG * T3 (vs. CG * T1)	3.17	2.73	−2.22	8.56	.25
TCMG * T2 (vs. CG * T1)	−2.52	2.48	−7.39	2.35	.31
TCMG * T3 (vs. CG * T1)	−0.37	2.67	−5.61	4.86	.89
**HbA1c**					
Intercept	5.73	0.15	5.44	6.01	<.001
Age	0.01	0.00	0.00	0.01	<.05
Male (vs. female)	−0.07	0.05	−0.16	0.02	.12
Group					
OMG (vs. CG)	−0.01	0.05	−0.12	0.09	.84
TCMG (vs. CG)	0.06	0.06	−0.05	0.17	.28
Time					
T2 (vs. T1)	−0.06	0.04	−0.14	0.01	.08
T3 (vs. T1)	−0.07	0.03	−0.14	−0.01	.04
*Group*Time*					
OMG * T2 (vs. CG * T1)	−0.06	0.05	−0.15	0.04	.27
OMG * T3 (vs. CG * T1)	−0.05	0.04	−0.14	0.04	.25
TCMG * T2 (vs. CG * T1)	−0.08	0.05	−0.18	0.02	.12
TCMG * T3 (vs. CG * T1)	−0.11	0.05	−0.21	−0.01	.03
***Yang*-deficiency body constitution**					
Intercept	33.24	2.67	28.01	38.47	<.001
Age	−0.10	0.04	−0.18	−0.02	.02
Male (vs. female)	−0.89	0.87	−2.58	0.81	.31
Group					
OMG (vs. CG)	0.36	1.28	−2.16	2.88	.78
TCMG (vs. CG)	3.24	1.30	0.69	5.79	.01
Time					
T2 (vs. T1)	−0.48	0.90	−2.24	1.28	.59
T3 (vs. T1)	−1.22	0.81	−2.81	0.38	.13
*Group*Time*					
OMG * T2 (vs. CG * T1)	-0.81	1.19	−3.16	1.53	.50

OMG * T3 (vs. CG * T1)	−0.46	1.16	−2.73	1.81	.69
TCMG * T2 (vs. CG * T1)	−3.15	1.50	−6.09	−0.21	.04
TCMG * T3 (vs. CG * T1)	−2.37	1.36	−5.04	0.29	.08
***Yin*-deficiency body constitution**					
Intercept	30.50	2.53	25.53	35.47	＜.001
Age	-0.09	0.04	-0.16	-0.01	.02
Male (vs. female)	0.25	0.87	-1.45	1.94	.78
Group					
OMG (vs. CG)	0.21	1.19	-2.13	2.55	.86
TCMG (vs. CG)	2.73	1.32	0.15	5.31	.04
Time					
T2 (vs. T1)	-0.02	0.95	-1.88	1.84	.98
T3 (vs. T1)	-1.07	0.73	-2.49	0.35	.14
*Group*Time*					
OMG * T2 (vs. CG * T1)	-0.02	1.30	-2.56	2.52	.99
OMG * T3 (vs. CG * T1)	0.03	1.07	-2.08	2.13	.98
TCMG * T2 (vs. CG * T1)	-2.29	1.52	-5.28	0.70	.13
TCMG * T3 (vs. CG * T1)	-1.62	1.32	-4.21	0.96	.22
**Phlegm-stasis body constitution**					
Intercept	27.99	2.54	22.01	31.98	<.001
Age	−0.08	0.04	−0.16	−0.01	.03
Male (vs. female)	−0.98	0.79	−2.54	0.57	.21
Group					
OMG (vs. CG)	1.12	1.24	−1.30	3.55	.36
TCMG (vs. CG)	3.24	1.22	0.86	5.63	.01
Time					
T2 (vs. T1)	0.84	1.02	−1.16	2.85	.41
T3 (vs. T1)	0.40	0.98	−1.53	2.33	.69
*Group*Time*					
OMG * T2 (vs. CG * T1)	−1.36	1.42	−4.16	1.44	.34
OMG * T3 (vs. CG * T1)	−1.88	1.28	−4.38	0.63	.14
TCMG * T2 (vs. CG * T1)	−3.45	1.55	−6.49	−0.42	.03
TCMG * T3 (vs. CG * T1)	−3.30	1.38	−6.01	−0.58	.02
**Body energy**					
Intercept	45.25	10.57	24.50	65.99	<.001
Age	−0.11	0.15	−0.41	0.19	.49
Male (vs. female)	4.76	2.72	−0.57	10.1	.08
Group					
OMG (vs. CG)	2.15	4.68	−7.03	11.32	.65
TCMG (vs. CG)	−1.66	4.60	−10.67	7.35	.72
Time					
T2 (vs. T1)	−1.15	3.97	−8.93	6.64	.77
T3 (vs. T1)	1.13	4.38	−7.48	9.73	.80
*Group*Time*					
OMG * T2 (vs. CG * T1)	-0.16	5.65	−11.23	10.91	.98
OMG * T3 (vs. CG * T1)	−5.84	6.02	−17.68	6.01	.33
TCMG * T2 (vs. CG * T1)	8.60	5.36	−1.91	19.11	.11
TCMG * T3 (vs. CG * T1)	7.81	5.69	−3.36	18.98	.17
**Physical component score**					
Intercept	54.18	3.41	47.50	60.86	<.001
Age	−0.08	0.06	−0.20	0.03	.15

Male (vs. female)	1.78	1.06	−0.29	3.86	.09
Group					
OMG (vs. CG)	−0.93	1.42	−3.71	1.85	.51
TCMG (vs.CG)	−2.52	1.66	−5.77	0.72	.13
Time					
T2 (vs. T1)	−1.05	1.10	−3.22	1.12	.34
T3 (vs. T1)	−0.07	1.04	−2.11	1.97	.95
*Group*Time*					
OMG * T2 (vs. CG * T1)	2.56	1.53	−0.44	5.56	.10
OMG * T3 (vs. CG * T1)	2.90	1.51	−0.07	5.89	.06
TCMG * T2 (vs. CG * T1)	4.93	1.51	1.97	7.89	.001
TCMG * T3 (vs. CG * T1)	4.89	1.52	1.92	7.87	.001
**Mental component score**					
Intercept	48.53	3.73	41.22	55.83	<.001
Age	0.01	0.05	−0.10	0.12	.84
Male (vs. female)	1.55	1.23	−0.87	3.97	.21
Group					
OMG (vs. CG)	−0.55	1.88	−4.23	3.12	.77
TCMG (vs. CG)	−3.77	1.86	−7.41	-0.13	.04
Time					
T2 (vs. T1)	−2.10	1.49	−5.01	0.82	.16
T3 (vs. T1)	−0.33	1.52	−3.36	2.71	.83
*Group*Time*					
OMG * T2 (vs. CG * T1)	4.63	1.89	0.91	8.35	.02
OMG * T3 (vs. CG * T1)	2.68	1.93	−1.16	6.51	.17
TCMG * T2 (vs. CG * T1)	8.11	2.18	3.82	12.40	<.001
TCMG * T3 (vs. CG * T1)	7.26	1.99	3.35	11.17	<.001
**BMI**					
Intercept	26.72	2.68	21.47	31.97	<.001
Age	−0.04	0.04	−0.12	0.05	.37
Male (vs. female)	1.51	0.75	0.03	2.99	.05
Group					
OMG (vs. CG)	1.01	0.86	−0.58	2.61	.24
TCMG (vs. CG)	0.64	0.87	−0.24	1.51	.47
Time					
T2 (vs. T1)	−0.21	0.11	−0.44	−0.01	.06
T3 (vs. T1)	−0.15	0.14	−0.43	0.13	.29
*Group*Time*					
OMG * T2 (vs. CG * T1)	−0.03	0.16	−0.34	0.29	.88
OMG * T3 (vs. CG * T1)	−0.24	0.18	−0.61	0.13	.19
TCMG * T2 (vs. CG * T1)	−0.12	0.16	−0.43	0.20	.47
TCMG * T3 (vs. CG * T1)	−0.37	0.18	−0.73	−0.02	.04
**DASH dietary behavior**					
Intercept	27.06	2.34	22.47	31.65	<.001
Age	0.18	0.04	0.10	0.25	<.001
Male (vs. female)	−1.83	0.75	−3.30	−0.36	.02
Group					
OMG (vs. CG)	−0.09	0.94	−1.94	1.76	.92
TCMG (vs. CG)	0.55	1.15	−1.71	2.80	.64
Time					
T2 (vs. T1)	1.57	0.67	0.25	2.89	.02

T3 (vs. T1)	2.07	0.77	0.57	3.57	.01
*Group*Time*					
OMG * T2 (vs. CG * T1)	0.22	0.84	−1.42	1.86	.79
OMG * T3 (vs. CG * T1)	−0.51	1.01	−2.49	1.48	.62
TCMG * T2 (vs. CG * T1)	1.21	0.94	−0.62	3.06	.19
TCMG * T3 (vs. CG * T1)	0.99	1.04	−1.05	3.04	.34
**Total physical activity**					
Intercept	283.61	581.26	−855.91	1423.14	.63
Age	5.28	9.08	−12.52	23.08	.56
Male (vs. female)	163.40	250.58	−327.75	654.54	.51
Group					
OMG (vs. CG)	676.72	313.80	61.81	1291.62	.03
TCMG (vs. CG)	−23.01	215.06	−444.52	398.51	.92
Time					
T2 (vs. T1)	181.50	129.01	−73.07	436.07	.16
T3 (vs. T1)	399.53	192.22	22.40	776.66	.04
*Group*Time*					
OMG * T2 (vs. CG * T1)	236.33	238.00	−231.76	704.43	.32
OMG * T3 (vs. CG * T1)	213.18	292.65	−360.50	786.86	.47
TCMG * T2 (vs. CG * T1)	248.59	223.99	−191.18	688.37	.27
TCMG * T3 (vs. CG * T1)	122.74	296.29	−459.22	704.71	.68

SE, standard error; CI, confidence interval; CG, control group; OMG, ordinary mHealth app group; TCMG, TCM mHealth app group; T1, baseline; T2, the end of intervention; T3, 1 month after the intervention.
