# Supplementary material for: The Effectiveness of a Traditional Chinese Medicine–Based Mobile Health App for Individuals With Prediabetes: Randomized Controlled Trial
Source: JMIR Mhealth Uhealth. 2023 Jun 20;11:e41099. doi: 10.2196/41099 (PMC10337399; doi:10.2196/41099)
Supplement: Multimedia Appendix 7 [file mhealth_v11i1e41099_app7.pdf]

Multimedia Appendix 7. Generalized estimating equation models to compare the outcomes between the TCM mHealth app (n=42) and ordinary mHealth app groups (n=41)

	β	SE	95%CI		*P*-value
			Lower	Upper	
**Fasting plasma glucose**					
Intercept	99.17	5.51	88.36	109.98	<.001
Age	0.11	0.09	−0.07	0.29	.24
Male (vs. female)	1.98	1.52	−1.01	4.96	.19
Group					
TCMG (vs. OMG)	1.26	1.84	−2.35	4.86	.49
Time					
T2 (vs. T1)	−1.41	1.59	−4.53	1.72	.38
T3 (vs. T1)	2.01	1.88	−1.69	5.71	.29
*Group*Time*					
TCMG * T2 (vs. OMG * T1)	−1.82	2.29	−6.30	2.66	.43
TCMG * T3 (vs. OMG * T1)	3.96	3.00	−9.91	1.99	.19
**HbA1c**					
Intercept	5.70	0.20	5.31	6.09	<.001
Age	0.01	0.003	0.001	0.01	.02
Male (vs. female)	−0.10	0.06	−0.21	0.01	.07
Group					
TCMG (vs. OMG)	−0.07	0.06	−0.19	0.05	.27
Time					
T2 (vs. T1)	−0.12	0.04	−0.19	−0.05	.001
T3 (vs. T1)	−0.18	0.03	−0.18	−0.06	<.001
*Group*Time*					
TCMG * T2 (vs. OMG * T1)	−0.02	0.05	−0.12	0.09	.78
TCMG * T3 (vs. OMG * T1)	−0.06	0.05	−0.15	0.04	.24
***Yang*-deficiency body constitution**					
Intercept	32.91	3.11	26.82	39.00	<.001
Age	−0.09	0.05	−0.18	0.01	.07
Male (vs. female)	−0.48	1.08	−2.59	1.63	.66
Group					
TCMG (vs. OMG)	2.93	1.35	0.28	5.58	.03
Time					
T2 (vs. T1)	−1.33	0.77	−2.86	0.19	.09
T3 (vs. T1)	−1.60	0.90	−3.37	0.18	.08
*Group*Time*					
TCMG * T2 (vs. OMG * T1)	−2.26	1.46	−5.12	0.61	.12
TCMG * T3 (vs. OMG * T1)	−1.96	1.46	−4.83	0.92	.18
***Yin*-deficiency body constitution**					
Intercept	29.19	3.12	23.07	35.32	＜.001
Age	−0.06	0.05	−0.16	0.03	.20
Male (vs. female)	0.61	1.15	−1.65	2.87	.60
Group					
TCMG (vs. OMG)	2.56	1.35	0.08	5.20	.06
Time					

T2 (vs. T1)	−0.23	0.88	−1.95	1.49	.79
T3 (vs. T1)	−0.94	0.82	−2.55	0.67	.25
*Group*Time*					
TCMG * T2 (vs. OMG * T1)	−2.16	1.45	-5.00	0.68	.14
TCMG * T3 (vs. OMG * T1)	−1.40	1.43	-4.20	1.41	.33
**Phlegm-stasis body constitution**					
Intercept	28.59	3.31	22.10	35.08	<.001
Age	−0.09	0.05	−0.19	0.01	.06
Male (vs. female)	−0.97	0.99	−2.91	0.96	.33
Group					
TCMG (vs. OMG)	2.13	1.32	−0.46	4.71	.11
Time					
T2 (vs. T1)	−0.35	0.96	−2.23	1.53	.71
T3 (vs. T1)	−1.47	0.85	−3.14	0.20	.08
*Group*Time*					
TCMG * T2 (vs. OMG * T1)	−.0.24	1.50	−5.19	0.70	.14
TCMG * T3 (vs. OMG * T1)	−1.55	1.27	−4.03	0.94	.22
**Body energy**					
Intercept	47.37	11.03	25.74	69.00	<.001
Age	−0.09	0.17	−0.42	0.23	.58
Male (vs. female)	3.36	3.23	−2.98	9.70	.30
Group					
TCMG (vs. OMG)	−4.00	4.34	−12.50	4.51	.36
Time					
T2 (vs. T1)	−0.86	4.16	−9.02	7.29	.84
T3 (vs. T1)	−3.95	4.12	−12.02	4.12	.34
*Group*Time*					
TCMG * T2 (vs. OMG * T1)	8.65	5.49	−2.11	19.41	.12
TCMG * T3 (vs. OMG * T1)	12.30	5.61	1.31	23.30	.03
**Physical component score**					
Intercept	49.39	3.87	41.81	56.97	<.001
Age	−0.01	0.06	−0.13	0.11	.84
Male (vs. female)	1.33	1.14	−0.92	3.57	.25
Group					
TCMG (vs. OMG)	−1.68	1.60	−4.81	1.45	.29
Time					
T2 (vs. T1)	1.94	1.03	−0.08	3.95	.06
T3 (vs. T1)	3.01	1.06	0.93	5.08	.01
*Group*Time*					
TCMG * T2 (vs. OMG * T1)	2.32	1.48	−0.58	5.22	.12
TCMG * T3 (vs. OMG * T1)	2.24	1.59	−0.87	5.35	.16
**Mental component score**					
Intercept	46.04	3.52	39.16	52.92	<.001
Age	0.05	0.06	−0.08	0.17	.47
Male (vs. female)	1.45	1.52	−1.53	4.43	.34
Group					
TCMG (vs. OMG)	−3.25	1.89	−6.96	0.46	.09
Time					
T2 (vs. T1)	2.64	1.07	0.53	4.75	.02
T3 (vs. T1)	2.26	1.17	−0.03	4.55	.05
*Group*Time*					

TCMG * T2 (vs. OMG * T1)	3.14	2.01	−0.82	7.10	.12
TCMG * T3 (vs. OMG * T1)	4.29	2.03	0.27	8.31	.04
**BMI**					
Intercept	28.25	3.94	20.53	35.97	<.001
Age	−0.05	0.06	−0.17	0.07	.44
Male (vs. female)	1.61	1.03	−0.40	3.62	.12
Group					
TCMG (vs. OMG)	1.61	1.03	−0.40	3.62	.12
Time					
T2 (vs. T1)	−0.24	0.11	−0.45	−0.03	.03
T3 (vs. T1)	−0.40	0.11	−0.63	−0.18	<.001
*Group*Time*					
TCMG * T2 (vs. OMG * T1)	−0.10	0.16	−0.41	0.21	.53
TCMG * T3 (vs. OMG * T1)	−0.14	0.18	−0.49	0.20	.41
**DASH dietary behavior**					
Intercept	23.98	2.91	18.28	29.69	<.001
Age	0.23	0.05	0.13	0.32	<.001
Male (vs. female)	−1.62	0.92	−3.42	0.18	.08
Group					
OMG (vs. TCMG)	0.65	1.05	−1.41	2.71	.54
Time					
T2 (vs. T1)	1.86	0.50	0.88	2.83	<.001
T3 (vs. T1)	1.61	0.64	0.35	2.88	.01
*Group*Time*					
TCMG * T2 (vs. OMG * T1)	1.06	0.79	−0.48	2.60	.18
TCMG * T3 (vs. OMG * T1)	1.44	0.96	−0.45	3.33	.14
**Total physical activity**					
Intercept	799.23	747.83	−666.66	2265.12	.29
Age	7.05	13.06	−18.55	32.64	.59
Male (vs. female)	281.57	345.12	−394.93	958.07	.42
Group					
OMG (vs. TCMG)	−684.94	330.15	−1332.02	−37.87	.04
Time					
T2 (vs. T1)	431.21	206.64	25.28	837.13	.04
T3 (vs. T1)	582.95	228.30	135.13	1030.78	.02
*Group*Time*					
TCMG * T2 (vs. OMG * T1)	4.99	275.49	−535.31	545.30	.99
TCMG * T3 (vs. OMG * T1)	−82.67	318.50	−708.14	542.80	.80

SE, standard error; CI, confidence interval; CG, control group; OMG, ordinary mHealth app group; TCMG, TCM mHealth app group; HbA1c, hemoglobin A1c; T1, baseline; T2, the end of intervention; T3, 1 month after the intervention.
